# Amino-acid-enriched cereals ready-to-use therapeutic foods (RUTF) are as effective as milk-based RUTF in recovering essential amino acid during the treatment of severe acute malnutrition in children: An individually randomized control trial in Malawi

**DOI:** 10.1371/journal.pone.0201686

**Published:** 2018-08-10

**Authors:** Wataru Sato, Chie Furuta, Keiko Matsunaga, Paluku Bahwere, Steve Collins, Kate Sadler, Peter Akomo, Chrissy Banda, Elizabeth Maganga, Sylvester Kathumba, Hitoshi Murakami

**Affiliations:** 1 Institute for Innovation, Ajinomoto Co., Inc. Tokyo, Japan; 2 Valid International, Oxford, United Kingdom; 3 Center for Epidemiology, Biostatistics, and Clinical Research, School of Public Health, Free University of Brussels, Brussels, Belgium; 4 Valid Nutrition, Cork, Republic of Ireland; 5 Ministry of Health Malawi, Lilongwe, Malawi; 6 Nutrition Improvement Department, Ajinomoto Co. Inc., Tokyo, Japan; TNO, NETHERLANDS

## Abstract

**Background:**

Ready-to-use therapeutic food (RUTF) is used to treat children suffering from severe acute malnutrition (SAM). Standard RUTF uses milk as the primary protein source, which makes the product expensive, and given the high worldwide SAM burden, having a less expensive effective alternative is a public health priority.

**Objective:**

The objective of this study was to evaluate whether newly developed amino acid-enriched milk-free RUTF (FSMS-RUTF) or amino acid-enriched low-milk RUTF (MSMS-RUTF) treatment could replenish plasma amino acids to levels comparable to those following standard peanut-milk RUTF (PM-RUTF) treatment and to improve understanding of the effects of treatment on anthropometric measurements. A secondary analysis was performed to test the noninferiority hypothesis of plasma essential amino acid (EAA) levels.

**Methods:**

Plasma EAA levels were measured in a nonblinded, 3-arm, parallel-group simple randomized controlled trial conducted in Malawi to examine the efficacy of FSMS-RUTF, MSMS-RUTF and PM-RUTF in the treatment of SAM in 2 groups of children aged 6–23 and 24–59 months (mo). Sample size calculations were performed based on the previous our study. A noninferiority margin was set at -25% of the PM-RUTF arm at discharge.

**Results:**

The relative values of the differences (95% CI) in plasma EAA levels between PM-RUTF treatment and FSMS-RUTF and MSMS-RUTF treatments at discharge were -7.9% (-18.6, 2.8) and 9.8% (0.2, 19.5), respectively, in children aged 6–23 mo, while in those aged 24–59 mo, the difference values were 17.8% (1.6, 34.1) and 13.6% (-2.8, 29.9), respectively.

**Conclusion:**

At discharge, the plasma EAA concentrations in 6-59-mo-old SAM children treated with FSMS-RUTF and MSMS-RUTF were not less than those of children treated with PM-RUTF. These findings indicate that treatment with either of the 3 RUTFs was associated with adequate protein synthesis and that all the formulations provided sufficient functional metabolites of plasma amino acids to support nutritional recovery from SAM.

## Introduction

Severe acute malnutrition (SAM) is classified by weight-for-height measurements being 3 standard deviations (SDs) below the median of the WHO growth curves, by the presence of bilateral pitting edema or by a mid-upper-arm circumference (MUAC) of less than 115 mm in children 6–59 months (mo) of age [1]. Ready-to-use therapeutic food (RUTF), a lipid-based and energy-dense paste, is the primary treatment for SAM.

The most widely used RUTF is peanut-milk RUTF (PM-RUTF) [2], which has been shown to be highly effective, but it is difficult to procure high-quality milk powder and peanut paste locally in developing countries. To lower costs, milk- and peanut-free RUTFs based on the locally available ingredients soy, maize, and sorghum (SMS) have been developed [3]. Initial studies of SMS-RUTF efficacy held in Zambia showed inconclusive results, and those in Democratic Republic of Congo (DRC) showed that SMS-RUTF was efficacious in children 24 mo or older but less so in children under 24 mo [3, 4]. In the latter study in particular, one possible explanation for the lower impact in the recovery of younger children was that the required levels of key amino acids involved in protein synthesis were not met since SMS-RUTF has a lower content of several amino acids [4]. To improve the efficacy, new RUTFs were developed with improved amino acid profiles: an amino acid-enriched milk-free SMS formulation (FSMS-RUTF) and an amino acid-enriched low-milk SMS formulation containing 9.3% (w/w) milk (MSMS-RUTF). An efficacy trial in Malawi showed positive results suggesting that both FSMS and MSMS-RUTF are efficacious in the treatment of SAM in children less than 5 years old [5].

Many efficacy trials for RUTFs have been implemented in the past, with positive outcomes in recovery of SAM and in recovery of anthropometric measurements [6, 7]. Further insights into the mechanistic properties of RUTF were mainly focused on the micronutrient nutrition, for which hemoglobin levels were often measured [8, 9]. However, analysis of macronutrients such as the plasma amino acid profile was not fully assessed in these RUTF efficacy trials because there are not yet sensitive biomarkers for assessing the state of protein malnutrition. However, the etiologies of kwashiorkor and marasmus, the two main types of SAM, are both associated with protein deficiencies; thus, investigation of the impact of RUTF treatments on the plasma amino acid profile in children recovering from SAM is useful for understanding nutritional recovery.

Recent studies have shown that changes in plasma amino acid profiles correlate with physiological variables such as dietary protein intake and malnutrition [4, 10, 11]. Plasma essential amino acids (EAAs; methionine, leucine, valine, isoleucine, lysine, phenylalanine, tryptophan, threonine and histidine) are reported to decrease in patients with SAM and recover after stabilization [10]. This recovery is thought to reflect the restoration of the proteogenic capacity leading to recovery from SAM. We believe that measuring plasma amino acids in children treated for SAM is very important for assessing the plasma amino acid profile of treated children with RUTFs. In particular, EAAs are extremely important in growing children because they are precursors for protein synthesis. Leucine is not only a precursor but also a key activating factor for protein synthesis *via* the mammalian target of rapamycin signaling pathway [12, 13]. Increased plasma EAA concentrations result in increased protein synthesis in human muscles [14].

Since the newly developed RUTFs are based on using plant proteins and supplementation with amino acids, comparing the protein nutrition of the treated children with that of PM-RUTF-treated children is important for understanding the biological mechanism by which the newly developed product affects SAM recovery. In a previous efficacy study of SMS-RUTF treatment conducted in DRC, we measured plasma amino acid levels in a subgroup of participating children [4]. In this study, we focused on the sulfur-containing amino acids since the plasma methionine concentration was decreased in the SMS-RUTF arm compared to the community controls and plasma cystine concentration was decreased in the SMS-RUTF arm compared to the PM-RUTF arm [4]. We refined the composition of FSMS-RUTF and MSMS-RUTF to enrich methionine because methionine is an indispensable amino acid and a portion of cysteine is derived from dietary methionine [15].

This study aimed to evaluate whether FSMS or MSMS-RUTF treatment could maintain plasma EAA, leucine, methionine and cystine to levels comparable to those in children treated with PM-RUTF and provide further understanding of the primary outcomes (FSMS- and MSMS-RUTF treatments showed noninferiority for recovery rate, program length of stay, and weight gain compared to PM-RUTF treatment) of the previously reported study [5]. A noninferiority hypothesis was assessed based on primary plasma EAA, leucine, methionine and cystine levels.

## Materials and methods

### Study design

This study is a secondary analysis of a nonblinded, 3-arm, parallel-group simple randomized controlled trial conducted in the central region of Malawi to examine the efficacy of FSMS-RUTF, MSMS-RUTF and PM-RUTF for the treatment of SAM in 2 groups of children aged 6–23 and 24–59 mo. This trial was registered at the Pan African Clinical Trials Registry as trial no. PACTR201505001101224 (http://www.pactr.org/ATMWeb/appmanager/atm/atmregistry?dar=true&tNo=PACTR201505001101224).

### Ethics

Before data collection began, we obtained permission to conduct the study from the National Ethics Committee of the Malawi Ministry of Health and from the Ajinomoto Institutional Review Board. At the time of admission, each child’s parent or caregiver was informed about the nature and purpose of the study and asked for verbal and written consent for their child to be included and for their child’s medical information to be used for research purposes. Given the short duration of the study, no interim analysis was planned, and no stopping rule was predefined. No serious side effects were detected, and no reasons for interrupting the study were identified.

### Setting

In mainstream treatments for SAM, children with no medical complications access treatment as outpatients while staying in their homes (community-based management of acute malnutrition; CMAM) rather than in therapeutic centers [16]. This trial was designed under the CMAM scheme and was carried out in 3 health districts in the central region of Malawi. A health district is divided into subadministrative areas, and 21 of these were selected for the study, with a community-based feeding center established in each to serve as daycare feeding centers. Participant recruitment into the study began in September 2015 and ended in June 2016. Treatment follow-up ended in August 2016.

Children with any medical or nutritional complications during follow-up were referred to the participating inpatient facility for appropriate treatment, after which they were readmitted into the daycare program and remained in their original study group. Children meeting referral criteria whose caregivers refused transfer and chose to remain in daycare were excluded from the study. Medical complications were defined using the WHO CMAM and Integrated Management of Childhood Illnesses standard definitions [17, 18]. Nutrition rehabilitation during inpatient treatment followed national guidelines, and therapeutic milks F-75 and F-100 were used as needed.

### Participant selection

Study participants were selected from all children aged 6–59 mo who had been diagnosed with SAM and admitted into the CMAM programs operated by the Ministry of Health. SAM was defined as a MUAC<115 mm or bilateral pitting edema of any degree. Children with an MUAC<115 mm and those with grade 1 or 2 bilateral pitting edema with good appetite and no medical complications were admitted directly into the daycare program and enrolled in the study.

There, they were re-examined by senior supervisors to confirm the SAM diagnosis. Children admitted into the CMAM program were excluded from the study if senior supervisors did not confirm the presence of SAM. Children with congenital or acquired disorders affecting growth, any history of any food allergy or intolerance or a history of treatment for SAM in the previous 3 mo and those from visiting families were also excluded.

This study used simple randomization, with each of the 21 sites recruiting subjects into each of the 3 arms at a ratio of 1:1:1. After confirming the subjects’ eligibility for study inclusion, we used a closed envelope method to randomly assign children to receive the FSMS-RUTF, MSMS-RUTF, or PM-RUTF treatment. The trial statistician prepared a computer-generated sequentially numbered randomization list that contained the allocations and codes for each site. These data were sent to the national study coordinator, who then assigned participants to groups at the time of enrollment. The required sample size was achieved in Jun 2018.

### Sample size

Sample size calculations were performed based on the DRC study [4] on key amino acids (EAA, leucine, methionine and cystine). The pooled SDs were 198.3, 30.2, 3.3 and 9.5 in EAA, leucine, methionine and cystine, respectively. Alpha was set at 0.05. The noinferiority margins were set at -25% which means for EAA, leucine, methionine and cystine noninferiority limit of 140.41 μM, 18.34 μM, 3.42 μM and 8.87 μM, and give sample size 25, 34, 12 and 15 patients per arm respectively to provide a power of at least 80%. These sample sizes were calculated using the “TwoSampleMean.NIS” function in the “TrialSize” (ver. 1.3) package for R (https://CRAN.R-project.org/package=TrialSize).

On the basis of the DRC study [4], we expected 20% loss to follow-up; thus, the total required sample sizes (2 subgroups) were 188, 255, 90 and 113 patients for EAA, leucine, methionine and cystine, respectively. This study was embedded in another study that required 1299 participants[5] and applied to children whose blood samples could be collected within 48 hours. This yielded in a sample size of 499, exceeding the minimum sample size required for this noninferiority study.

### Treatment protocol

The treatment was conducted based on a general CMAM program. Children staying within 1 h walking distance from feeding points in the study community were enrolled and followed-up from admission to nutrition recovery using a daycare approach. Enrolled children attended the site daily from 9 a.m. to 3 p.m. Children were offered 200 kcal/kg/day of one of the study RUTFs. The nutritional compositions of the study RUTFs (FSMS, MSMS and PM) are shown in S1 and S2 Tables. Children were declared nonrecovered if they did not meet the discharge criteria (MUAC≥ 12.5 cm and no edema) after 3 consecutive mo of treatment. Apart from the difference in RUTF administered, children of all three study groups were treated and given medical treatment generally following Malawi national guidelines, with the exception of the daycare approach.

### Noninferiority margin assumption

To date, there are no official clinical values for the concentrations of plasma amino acids. Therefore, in order to define a margin for the noninferiority analysis, we used circadian values of plasma amino acids in healthy adult humans since plasma amino acid levels are known to exhibit diurnal variations. Studies have shown that the concentrations of most plasma amino acids oscillate more than 25% during the day [19, 20]. This indicates that the largest clinically acceptable difference in plasma amino acid concentrations exceeds 25%. Furthermore, the difference in plasma amino acid concentrations between healthy infants and those with severe malnutrition is more than 35% on average [21]. Thus, we decided to set the noninferiority margin at the most conservative value of -25% of the control (PM-RUTF).

### Sample collection

**Fig 1:**
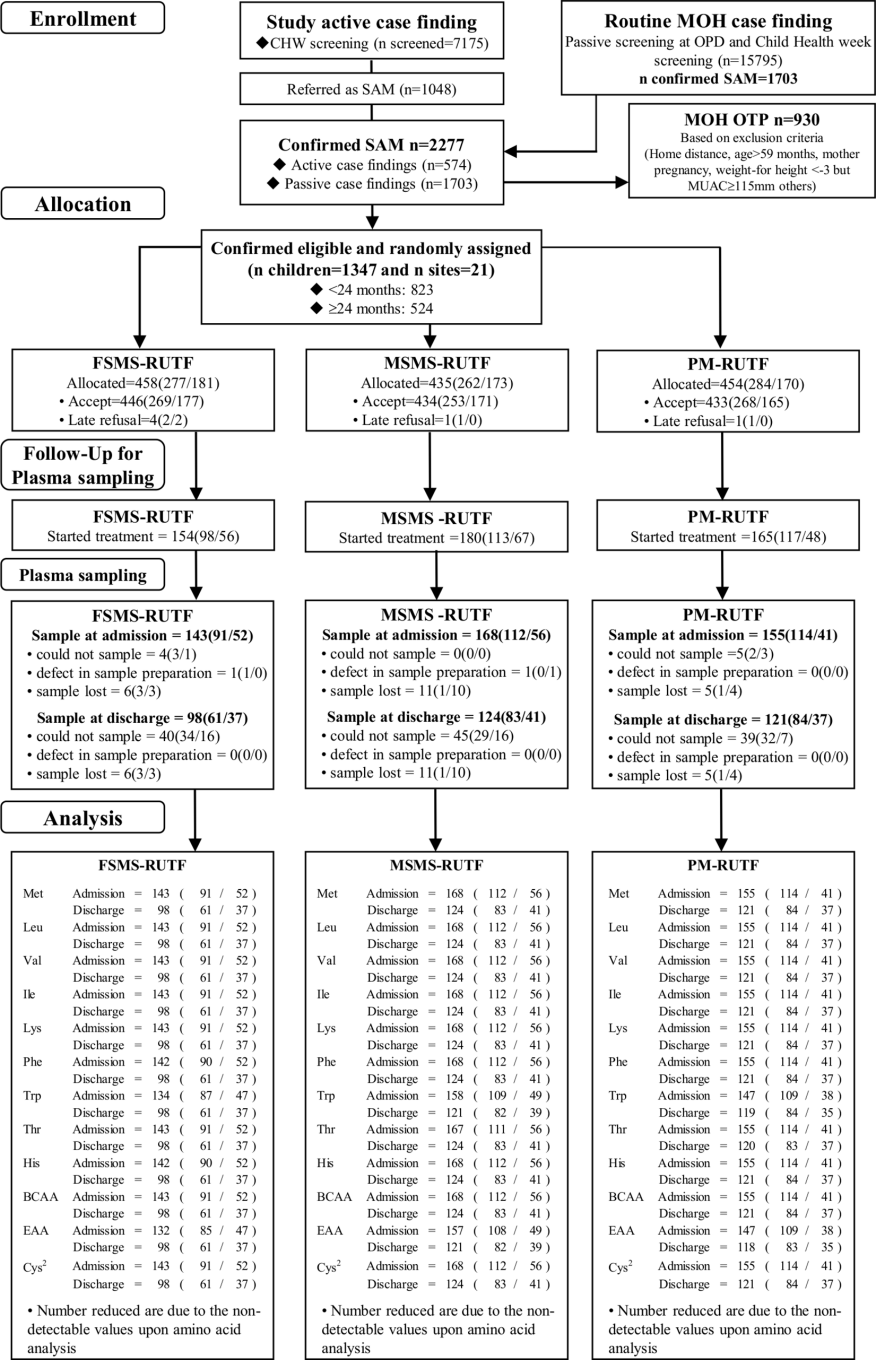
Study participant flow diagram. CHW, community health worker; MOH, Ministry of Health; OPD, outpatient department; OTP, outpatient program; SAM, severe acute malnutrition; MUAC, mid-upper arm circumference; FSMS, milk-free, soy, maize, and sorghum; RUTF, ready-to-use therapeutic food; MSMS, milk, soy, maize, and sorghum; PM, peanut and milk; Met, methionine; Leu, leucine; Val, valine; Ile, isoleucine; Lys, lysine; Phe, phenylalanine; Trp, tryptophan; Thr, threonine; His, histidine; BCAAs, branched-chain amino acids; EAAs, essential amino acids; Cys2, cystine.

Blood sampling for amino acid analysis was performed on the subjects from whom blood samples could be collected within 48 h after enrollment, and collections continued until the planned sample number was reached (n = 499). The flow diagram of the participants is indicated in Fig 1. Trained pediatric phlebotomists collected venous blood and obtained plasma using ethylenediaminetetraacetic acid disodium dehydrate (CAS: 6381-92-6) as an anticoagulant. All blood specimens were collected in the morning, immediately stored in a CubeCooler (Forte Grow Medical Co. Ltd., Tochigi, Japan) and kept at 4°C [22]. Plasma was separated within 24 h, mixed with 2 volumes of 5% (w/w) trichloroacetic acid, and centrifuged immediately (4°C, 20 min, 8000 × g) to remove the precipitated protein. Samples were stored at -80°C until they were shipped to Japan for the measurement of amino acid concentrations. During transportation, the samples were kept frozen in cooler boxes with dry ice. The samples were quality checked and handled were done by an internal standard method by using s-(2-aminoethyl)-l-cystein HCl (CAS: 4099-35-8).

### Amino acid analysis

Amino acid (methionine, leucine, valine, isoleucine, lysine, phenylalanine, tryptophan, threonine, histidine and cystine) concentrations were measured by an automatic amino acid analyzer (L-8800; Hitachi High-Technologies Corporation., Tokyo, Japan). Briefly, amino acids, separated by cation-exchange chromatography, were detected spectrophotometrically after a postcolumn reaction with ninhydrin reagent.

Total BCAA (leucine, valine and isoleucine) and EAA concentrations were calculated as the summation of the concentration of each BCAA and each EAA. Plasma amino acid concentrations are given in units of micromolar (μM).

### Data analysis

All analyses were performed on an intention-to-treat basis. Means and SDs or standard errors (SEs) or proportions and 95% confidence intervals (CIs) were used to describe the admission, discharge and difference parameters, as appropriate. To adjust for population heterogeneity, we divided the children into two subgroups, a 6-23-mo-old subgroup and a 24-59-mo-old subgroup, as described the study protocol (S1 Study Protocol). Tukey’s multiple comparisons tests or Fisher’s exact test were used for baseline characteristic comparisons.

Plasma amino acids analysis considered the CMAM program by using mixed models that recognized the multilevel structure of the data where individual patients were nested within one of the 21 clusters. A model-based approach can be efficient and effective for handling missing data [23]. Hence, we used a linear mixed-effects model (LMM) to predict plasma amino acid concentrations at admission and discharge. Fixed effects in the model included the intervention (SMS, MSMS and PM RUTFs), sampling point (admission or discharge) and their interaction. The model was adjusted for individual admission values and the random effects of subadministrative areas. The following generic model was run for each amino acid:

$${Y=\beta }_{0(\mathrm{intercept})}{+\beta }_{1(\mathrm{intervention})}{+\beta }_{2(\mathrm{sampling}\;\mathrm{point})}{+\beta }_{3(\mathrm{intervention}\;x\;\mathrm{sampling}\;\mathrm{point})}{+\beta }_{4(\mathrm{admission}\;\mathrm{value})}{+r}_{\left(\mathrm{random}\;\mathrm{effect}\right)}$$

Since intervention had not yet started at admission, the “intervention” variable included the allocation effect of each arm (FSMS arm, MSMS arm and PM arm); the “sampling point” variable included the effect of a treatment period common to the arms. Interaction of “intervention” and “sampling point” included the effect of each RUTF (FSMS, MSMS and PM). The “admission value” variable included the effect of interindividual differences in plasma amino acid concentrations. To determine the model coefficients, the model was fitted by a residual maximum likelihood estimation using age subgroup data with the “lme4” (ver. 1.1.13) package for R [24]. Estimated coefficients were used for the prediction of amino acid concentrations or statistical tests.

The calculated predicted plasma amino acid concentrations at discharge were tested in a noninferiority hypothesis that those of FSMS-RUTF and MSMS-RUTF would not be less than those of PM-RUTF. Our generic models were adjusted for individual admission value; therefore, predicted discharge values were controlled by each subject admission value, and comparison of predicted discharge values between arms was equal to comparison of actual differences between admission and discharge between arms. For the noninferiority test, the 2-sided 95% CI of the differences between the FSMS-RUTF arm and the PM-RUTF arm and between the MSMS-RUTF arm and the PM-RUTF arm were estimated by simultaneous inference procedures in the LMM with the “multcomp” (ver. 1.4.6) package for R [25]. Estimated differences are shown as relative values based on the plasma amino acid concentration of the PM-RUTF arm at discharge.

All statistical analyses were performed within the R (ver. 3.3.0) platform (https://www.r-project.org/).

## Results

### Baseline results

Table 1 presents the baseline characteristics of the children included in the analyses for each age subgroup. In the study, marasmus (without edema) was the dominant form of SAM among children aged 6–23 mo, and kwashiorkor (with edema) was the dominant form of SAM among children aged 24–59 mo. Percentage of sex significantly differed between the MSMS-RUTF arm and PM-RUTF arm for children aged 24–59 mo. MUAC and height were significantly different between the FSMS-RUTF arm and PM-RUTF arm for children aged 24–59 mo. There were no significant differences (p>0.05) between the PM-RUTF arm and the MSMS-RUTF and FSMS-RUTF arms in the other baseline parameters considered in either of the 2 age subgroups. Breastfeeding rate was not significantly different between arms in either of the 2 age subgroups (data not shown).

**Table 1:** Baseline characteristics for children^1^.

		6–23 mo of age												24–59 mo of age											
Criteria		FSMS-RUTF				MSMS-RUTF				PM-RUTF				FSMS-RUTF				MSMS-RUTF				PM-RUTF			
Participants, n		91				112				114				52				56				41			
All																									
	Male sex, n	45	(	49.5%	)	50	(	44.6%	)	51	(	44.7%	)	27	(	51.9%	)	25	(	44.6%	)	30	(	73.2%	)
	Age, mo	13.5	±	4.6		14.2	±	5.9		13.9	±	4.9		30.4	±	5.6		31.9	±	7.2		33.7	±	8.3	
	Midupper arm circumference, mm	111	±	9		114	±	9		113	±	9		120	±	14		128	±	16		128	±	16	
	Weight, kg	6.3	±	1.1		6.6	±	1.4		6.5	±	1.2		8.8	±	2.0		9.8	±	1.9		9.6	±	2.4	
	Height, cm	67.3	±	6.1		68.3	±	6.3		67.7	±	5.9		78.1	±	6.1		81.9	±	6.5		81.5	±	6.6	
	Bilateral pitting edema	30	(	33.0%	)	42	(	37.5%	)	33	(	28.9%	)	30	(	57.7%	)	44	(	78.6%	)	30	(	73.2%	)
	Weight-for-age z score	-3.5	±	1.3		-3.2	±	1.2		-3.4	±	1.2		-3.4	±	1.5		-2.6	±	1.5		-3.1	±	1.6	
	Height-for-age z score	-3.4	±	1.7		-3.2	±	1.4		-3.4	±	1.6		-3.8	±	1.4		-2.9	±	1.7		-3.4	±	1.5	
	Weight-for-height z score	-2.3	±	1.3		-2.0	±	1.2		-2.1	±	1.2		-1.9	±	1.5		-1.4	±	1.4		-1.9	±	1.8	
Children without edema, n		61				70				81				22				12				11			
	Male sex, n	31	(	50.8%	)	35	(	50.0%	)	36	(	44.4%	)	9	(	40.9%	)	4	(	33.3%	)	9	(	81.8%	)
	Age, mo	12.2	±	4.7		12.8	±	5.6		13.1	±	4.5		29.1	±	5.6		31.9	±	6.3		32.0	±	7.4	
	Midupper arm circumference, mm	108	±	7		109	±	6		110	±	5		110	±	4		108	±	7		110	±	4	
	Weight, kg	5.9	±	0.9		6.0	±	1.0		6.1	±	1.0		7.3	±	0.9		8.0	±	1.0		7.7	±	1.2	
	Height, cm	65.4	±	5.9		66.0	±	5.4		66.6	±	5.6		74.6	±	4.5		78.7	±	5.0		77.5	±	5.3	
	Weight-for-age z score	-3.8	±	1.2		-3.8	±	1.0		-3.6	±	1.1		-4.5	±	0.7		-4.0	±	0.9		-4.5	±	0.9	
	Height-for-age z score	-3.6	±	1.9		-3.6	±	1.4		-3.5	±	1.6		-4.6	±	1.1		-3.8	±	1.3		-4.3	±	1.4	
	Weight-for-height z score	-2.4	±	1.2		-2.4	±	1.1		-2.3	±	1.1		-3.0	±	1.0		-2.8	±	0.9		-3.4	±	0.9	
Children with edema, n		30				42				33				30				44				30			
	Male sex, n	14	(	46.7%	)	15	(	35.7%	)	15	(	45.5%	)	18	(	60.0%	)	21	(	47.7%	)	21	(	70.0%	)
	Age, mo	16.1	±	3.2		16.4	±	5.7		15.9	±	5.1		31.3	±	5.5		32.0	±	7.5		34.4	±	8.7	
	Midupper arm circumference, mm	118	±	10		121	±	9		120	±	11		128	±	13		134	±	13		134	±	14	
	Weight, kg	7.2	±	1.2		7.7	±	1.2		7.2	±	1.3		9.8	±	1.9		10.2	±	1.8		10.3	±	2.4	
	Height, cm	71.2	±	4.3		72.1	±	6.0		70.3	±	5.8		80.7	±	5.8		82.7	±	6.6		83.0	±	6.5	
	Weight-for-age z score	-3.1	±	1.3		-2.4	±	1.0		-2.9	±	1.3		-2.6	±	1.4		-2.2	±	1.4		-2.6	±	1.5	
	Height-for-age z score	-3.0	±	1.1		-2.6	±	1.1		-3.1	±	1.5		-3.3	±	1.3		-2.7	±	1.8		-3.1	±	1.4	
	Weight-for-height z score	-2.1	±	1.4		-1.4	±	1.1		-1.7	±	1.1		-1.1	±	1.3		-1.0	±	1.3		-1.3	±	1.7	

^1^Values are expressed as n (%) or means ± SDs unless otherwise indicated.

FSMS, milk-free, soy, maize, and sorghum; MSMS, milk, soy, maize, and sorghum; PM, peanut and milk; RUTF, ready-to-use therapeutic food.

Table 2 presents the predicted plasma amino acid concentrations at admission and discharge in each age subgroup. For all amino acids, the β_1(intervention)_ coefficients of each intervention (FSMS-RUTF, MSMS-RUTF and PM-RUTF) in the model were not significant. As described in the methods section, “intervention” variable included the allocation effect of each arm (FSMS, MSMS and PM). This indicated that there was no allocation bias regarding amino acid concentration in the FSMS-RUTF, MSMS-RUTF and PM-RUTF arms at admission.

**Table 2:** Predicted plasma amino acid concentrations in the age subgroup^1^.

	6–23 mo of age																		24–59 mo of age																	
	FSMS-RUTF						MSMS-RUTF						PM-RUTF						FSMS-RUTF						MSMS-RUTF						PM-RUTF					
Amino acids	Admission^*^			Discharge			Admission^*^			Discharge			Admission^*^			Discharge			Admission^*^			Discharge			Admission^*^			Discharge			Admission^*^			Discharge		
Methionine	22.5	±	1.0	23.5	±	1.2	23.3	±	0.9	26.6	±	1.0	22.8	±	0.9	26.2	±	1.0	22.9	±	1.3	24.6	±	1.5	21.6	±	1.2	28.6	±	1.5	22.2	±	1.5	23.9	±	1.6
Leucine	112.4	±	3.7	119.5	±	4.7	112.2	±	3.3	137.3	±	3.9	116.0	±	3.3	125.4	±	3.9	109.2	±	5.7	153.3	±	6.8	104.2	±	5.5	146.0	±	6.6	108.5	±	6.4	114.5	±	7.0
Valine	175.3	±	6.2	179.7	±	7.9	174.5	±	5.6	215.2	±	6.5	183.0	±	5.6	210.6	±	6.6	168.5	±	9.4	230.7	±	11.2	158.0	±	9.1	224.6	±	10.8	167.5	±	10.5	185.3	±	11.6
Isoleucine	74.8	±	2.7	70.8	±	3.4	73.4	±	2.4	84.6	±	2.8	74.0	±	2.4	77.8	±	2.9	75.9	±	4.2	90.9	±	5.0	68.9	±	4.0	88.2	±	4.7	71.3	±	4.6	69.8	±	5.1
Lysine	188.2	±	8.0	179.4	±	10.0	186.7	±	7.3	224.2	±	8.4	175.6	±	7.2	191.4	±	8.4	187.9	±	10.4	223.9	±	12.4	175.7	±	9.9	224.6	±	11.8	172.3	±	11.7	184.8	±	12.8
Phenylalanine	74.4	±	2.2	66.1	±	2.8	73.0	±	2.0	76.7	±	2.4	74.3	±	2.0	77.4	±	2.4	80.1	±	3.6	82.5	±	4.3	74.7	±	3.5	89.2	±	4.1	74.9	±	4.1	76.6	±	4.5
Tryptophan	24.4	±	1.1	25.6	±	1.4	23.8	±	1.0	31.9	±	1.2	25.3	±	1.0	29.7	±	1.2	17.9	±	1.6	27.4	±	1.9	15.8	±	1.6	28.9	±	1.9	15.9	±	1.8	26.9	±	2.1
Threonine	87.1	±	3.2	82.9	±	4.0	87.0	±	2.9	91.7	±	3.3	91.6	±	2.8	95.2	±	3.4	84.1	±	4.3	94.5	±	5.2	77.6	±	4.2	88.4	±	5.0	84.5	±	4.9	85.2	±	5.4
Histidine	70.6	±	1.7	69.0	±	2.1	70.6	±	1.5	73.8	±	1.8	71.8	±	1.5	73.2	±	1.8	75.8	±	2.4	80.6	±	2.9	74.5	±	2.4	76.2	±	2.8	78.2	±	2.8	65.9	±	3.0
BCAA	362.6	±	12.4	370.0	±	15.6	360.1	±	11.1	437.1	±	12.9	373.0	±	11.1	413.7	±	13.1	353.4	±	18.7	474.9	±	22.4	331.2	±	18.1	459.1	±	21.5	347.1	±	21.0	369.4	±	23.0
EAA	841.5	±	26.3	823.1	±	32.9	834.7	±	23.3	981.6	±	27.2	848.4	±	23.2	893.6	±	27.6	864.6	±	37.4	1030.4	±	43.6	812.5	±	36.5	993.7	±	44.6	825.2	±	41.2	874.8	±	47.2
Cystine	22.6	±	0.6	26.9	±	0.8	22.5	±	0.6	27.9	±	0.7	24.1	±	0.6	29.5	±	0.7	20.5	±	0.9	29.7	±	1.1	19.7	±	0.9	28.5	±	1.0	20.7	±	1.0	30.2	±	1.1

^1^ Plasma amino acid concentrations were predicted from the mixed model by using the age subgroup data. The fixed effects were intervention (RUTF) and sampling point (admission or discharge). The model was adjusted for admission value and random effect of subadministrative areas. Predicted concentrations were expressed as the means ± SEs [μM].

^*^ There were no significant differences between the plasma amino acid concentrations of the FSMS-RUTF or MSMS-RUTF arms and those of the PM-RUTF arm at admission.

FSMS, milk-free, soy, maize, and sorghum; MSMS, milk, soy, maize, and sorghum; PM, peanut and milk; RUTF, ready-to-use therapeutic food; BCAA, branched-chain amino acid; EAA, essential amino acid.

### Noninferiority analyses of plasma amino acids in the age subgroups

**Fig 2:**
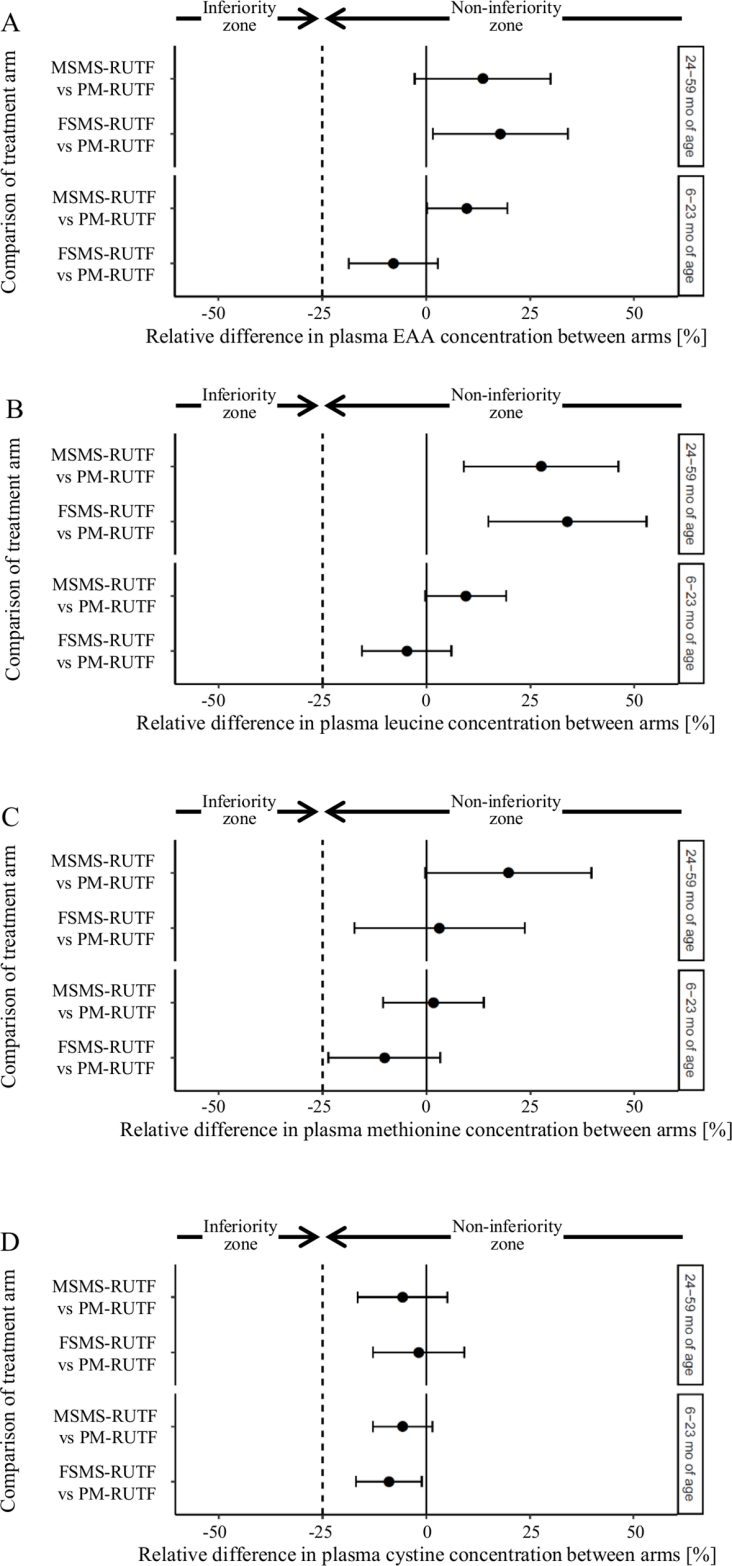
Comparison of the difference in plasma amino acid concentrations at discharge between the FSMS-RUTF arm and PM-RUTF arm and between the MSMS-RUTF arm and PM-RUTF arm by using the age subgroup data. The differences are shown as relative values based on the plasma amino acid concentration of the PM-RUTF arm at discharge. 95% CIs were estimated by simultaneous inference procedures in the mixed model. The filled circle and error bar indicate a point estimate of the difference and 95% CI. The dotted line shows the noninferiority margin (-25%). A: Relative difference in plasma EAA concentration between arms. B: Relative difference in plasma leucine concentration between arms. C: Relative difference in plasma methionine concentration between arms. D: Relative difference in plasma cystine concentration between arms.

For children aged 6–23 mo, the relative values of the difference (95% CI) in plasma EAA concentration based on the PM-RUTF arm at discharge were -7.9% (-18.6, 2.8) and 9.8% (0.2, 19.5) for the FSMS-RUTF and MSMS-RUTF arms, respectively (Fig 2A, Table 3). In those aged 24–59 mo, the relative values of the difference (95% CI) in the EAA concentration based on PM-RUTF arm at discharge were 17.8% (1.6, 34.1) and 13.6% (-2.8, 29.9) for the FSMS-RUTF and MSMS-RUTF arms, respectively (Fig 2A, Table 3). Absolute values are shown in S3 Table. These results indicated that the plasma EAA concentrations of the FSMS-RUTF and MSMS-RUTF arms were not less than those of the PM-RUTF arm at discharge in both age groups (Fig 2A, Table 3).

**Table 3:** Testing noninferiority of the plasma amino acid concentrations at discharge between the PM-RUTF arm and the FSMS-RUTF and MSMS-RUTF arms in the age subgroup^1^.

		6–23 mo of age						24–59 mo of age					
Amino acids	Comparison of treatment arm	Difference [%]	(95% CI)^2^					Difference [%]	(95% CI)^2^				
Methionine	FSMS-RUTF vs PM-RUTF	-10.1	(	-23.6	,	3.3	)	3.1	(	-17.3	,	23.6	)
	MSMS-RUTF vs PM-RUTF	1.7	(	-10.5	,	13.8	)	19.7	(	-0.3	,	39.7	)
Leucine	FSMS-RUTF vs PM-RUTF	-4.7	(	-15.5	,	6.0	)	33.9	(	14.9	,	52.9	)
	MSMS-RUTF vs PM-RUTF	9.5	(	-0.3	,	19.2	)	27.6	(	9.0	,	46.2	)
Valine	FSMS-RUTF vs PM-RUTF	-14.7	(	-25.5	,	-3.8	)	24.5	(	5.3	,	43.7	)
	MSMS-RUTF vs PM-RUTF	2.2	(	-7.6	,	12.0	)	21.3	(	2.3	,	40.2	)
Isoleucine	FSMS-RUTF vs PM-RUTF	-9.0	(	-21.7	,	3.7	)	30.2	(	7.7	,	52.7	)
	MSMS-RUTF vs PM-RUTF	8.8	(	-2.7	,	20.3	)	26.4	(	4.4	,	48.3	)
Lysine	FSMS-RUTF vs PM-RUTF	-6.3	(	-21.2	,	8.6	)	21.2	(	-0.2	,	42.5	)
	MSMS-RUTF vs PM-RUTF	17.1	(	3.7	,	30.6	)	21.5	(	0.8	,	42.3	)
Phenylalanine	FSMS-RUTF vs PM-RUTF	-14.7	(	-25.1	,	-4.3	)	7.7	(	-10.2	,	25.6	)
	MSMS-RUTF vs PM-RUTF	-0.9	(	-10.3	,	8.4	)	16.4	(	-1.0	,	33.9	)
Tryptophan	FSMS-RUTF vs PM-RUTF	-13.6	(	-27.6	,	0.4	)	1.9	(	-20.7	,	24.4	)
	MSMS-RUTF vs PM-RUTF	7.5	(	-5.3	,	20.2	)	7.6	(	-15.1	,	30.3	)
Threonine	FSMS-RUTF vs PM-RUTF	-12.9	(	-25.1	,	-0.8	)	11.0	(	-8.3	,	30.4	)
	MSMS-RUTF vs PM-RUTF	-3.7	(	-14.7	,	7.3	)	3.8	(	-15.2	,	22.8	)
Histidine	FSMS-RUTF vs PM-RUTF	-5.7	(	-14.1	,	2.7	)	22.3	(	8.2	,	36.4	)
	MSMS-RUTF vs PM-RUTF	0.9	(	-6.7	,	8.5	)	15.6	(	1.8	,	29.6	)
BCAA	FSMS-RUTF vs PM-RUTF	-10.6	(	-21.5	,	0.3	)	28.5	(	9.4	,	47.6	)
	MSMS-RUTF vs PM-RUTF	5.6	(	-4.2	,	15.5	)	24.3	(	5.4	,	43.0	)
EAA	FSMS-RUTF vs PM-RUTF	-7.9	(	-18.6	,	2.8	)	17.8	(	1.6	,	34.1	)
	MSMS-RUTF vs PM-RUTF	9.8	(	0.2	,	19.5	)	13.6	(	-2.8	,	29.9	)
Cystine	FSMS-RUTF vs PM-RUTF	-9.0	(	-16.9	,	-1.1	)	-1.8	(	-12.8	,	9.2	)
	MSMS-RUTF vs PM-RUTF	-5.7	(	-12.9	,	1.5	)	-5.7	(	-16.5	,	5.1	)

^1^ The point estimate and 95% CI of the difference in plasma amino acid concentrations at discharge between the FSMS-RUTF and PM-RUTF arms and between the MSMS-RUTF and PM-RUTF arms by using the age subgroup data. The differences are shown as relative values based on the plasma amino acid concentration of the PM-RUTF arm at discharge.

^2^ CIs were estimated by simultaneous inference procedures in mixed model. The noninferiority margin was -25% of the plasma amino acid concentration of the PM-RUTF arm at discharge. Therefore, the lower limit of 95% CI larger than -25% indicated noninferiority.

FSMS, milk-free, soy, maize, and sorghum; MSMS, milk, soy, maize, and sorghum; PM, peanut and milk; RUTF, ready-to-use therapeutic food; CI, confidence interval; BCAA, branched-chain amino acid; EAA, essential amino acid.

Similarly, the plasma concentrations of leucine in the FSMS-RUTF and MSMS-RUTF arms were also not less than the concentration in the PM-RUTF arm in both age groups (Fig 2B, Table 3).

For children aged 6–23 mo, the relative values of the difference (95% CI) in plasma methionine concentration based on the PM-RUTF arm at discharge were -10.1% (-23.6, 3.3) and 1.7% (-10.5, 13.8) for the FSMS-RUTF and MSMS-RUTF arms, respectively (Fig 2C, Table 3). In those aged 24–59 mo, the relative values of the difference (95% CI) in the methionine concentration based on the PM-RUTF arm at discharge was 3.1% (-17.3, 23.6) and 19.7% (-0.3, 39.7) for the FSMS-RUTF and MSMS-RUTF arms, respectively (Fig 2C, Table 3). Absolute values are shown in S3 Table. These results indicated that the plasma methionine concentrations in the FSMS-RUTF and MSMS-RUTF arms were not less than those in the PM-RUTF arm at discharge in both age groups (Fig 2C, Table 3).

Similarly, the plasma concentrations of cystine in the FSMS-RUTF and MSMS-RUTF arms were also not less than those in the PM-RUTF arm in both age groups (Fig 2D, Table 3).

## Discussion

In this report, we focused on assessing both EAAs and leucine to compare the efficacy of FSMS-RUTF and MSMS-RUTF with that of PM-RUTF in the noninferiority test since they EAAs and leucine are the key amino acids in protein synthesis. Our hypothesis was that maintaining an adequate concentration of plasma EAAs is important for protein synthesis. The noninferiority of the differences in plasma EAA and leucine concentrations in the FSMS–RUTF arm and the MSMS–RUTF arm at discharge (Fig 2, Table 3) demonstrated comparable levels of protein synthesis, which explains the reported SAM recovery rates (percentage of children who were discharged as recovered from the study divided by the total number of children who exited the study) [5].

The protein synthesis rate is higher in infants and young children and decreases with aging [26–28]. This pattern is also supported by the protein requirements of infants and children, which are reported to be highest among 0.5-year-olds (1.12 g protein/kg body weight/day), with a decrease and plateau among 3-year-olds (0.73 g protein/ kg body weight/day) [19]. Therefore, especially in young children in which the protein synthesis rate and requirements are high, if an adequate supply of proteins and amino acids is not provided, muscle tissue formation and albumin synthesis will be inhibited. Therefore, adequate protein and amino acid nutrition in children with SAM results in muscle tissue accretion and albumin synthesis, resulting in improvements in MUAC and the absence of bilateral pitting edema, which are clinical criteria of recovery from SAM. Apparently, the amounts of proteins and amino acids supplied from FSMS-RUTF and MSMS-RUTF are adequate for the recovery of plasma EAA and leucine to levels similar to those of PM-RUTFs and are, hence, adequate for recovery from SAM.

Our second aim was to verify whether the enriched concentrations of sulfur-containing amino acids in plasma in the FSMS-RUTF and MSMS-RUTF treatments were fully maintained compared to those in the PM-RUTF treatment. The results from this study revealed that plasma methionine and cystine concentrations in the FSMS–RUTF arm and the MSMS–RUTF arm were not less than those in the PM-RUTF arm at discharge (Fig 2, Table 3). This finding strongly suggests that the FSMS-RUTF treatment and MSMS-RUTF treatment could supply adequate amounts of methionine and cystine for children recovering from SAM.

Glutathione (GSH) is thought to play an important role in recovery from malnutrition, especially in kwashiorkor. GSH is considered to be the most abundant molecule among the endogenous antioxidants, and the GSH redox cycle is a major component of the body’s overall antioxidant defenses. GSH synthesis is largely limited by the availability of its precursor L-cysteine, and a portion of it is derived from methionine [15]. In children with kwashiorkor, GSH concentration in the blood and body organs is lower than in healthy subjects [29, 30]. Another study showed that the GSH concentration increased in patients who recovered from malnutrition and decreased in patients who did not [31]. These articles strongly support the idea that oxidative stress plays an important role in the pathophysiology of malnutrition. Several biomarkers of oxidant-induced lipid peroxidation are increased in children with SAM [32, 33]. Therefore, the noninferior results of plasma methionine and cystine in the FSMS-RUTF and MSMS-RUTF treatments of this study suggest that one mechanism underlying recovery from malnutrition under treatment with FSMS-RUTF and MSFS-RUTF might be the achievement of a stable supply of GSH through the maintenance of plasma methionine and cystine levels in the treatment arms.

One of the major limitations of this study is that we could not control for the mealtime of the subjects upon blood draw. Since concentrations of plasma amino acids are known to be influenced by nutrient intake during meals, the ideal blood draw for detecting baseline levels of plasma amino acids would be after a sufficient time intervals after the last meal [34]. Due to the fact that most of the study participants were breastfed and required frequent feeding yet were severely malnourished, we could not enforce the fasting of the children. These subjects’ background might theoretically limit the generalizability of our findings. However, baseline comparison of breastfeeding rate showed no significant difference between arms. This result indicates that the condition is equal in the populations of the three arms. Thus, the results may be generalized even the study population is dominantly breastfed.

Another major limitation of the study was the unavailability of samples at the time of discharge. Data at discharge were missing for children who defaulted or had died. The proportion of missing data due to default was large, and that due to death was very small, as previously described [5]. Default occurred randomly in the interventions and randomly affected the plasma amino acid concentrations. Hence, we believe that the reasons for missing data are mostly MCAR (missing completely at random). The National Research Council recommends a model approach for treating missing data in the report [23]. Therefore, we believe that our model analysis considers missing data and might generalize the unavailability of samples at discharge.

The present clinical trial in Malawi showed a noninferiority of FSMS-RUTF and MSMS-RUTF compared to PM-RUTF for children aged 6–59 mo in the recovery rate and weight gain reported in a previously published paper [5] and the results of plasma EAA concentrations presented in this paper. These results clearly demonstrate that the protein source of the RUTF does not influence the effectiveness of the product if amino acids are well balanced. This finding is important not only scientifically but also in terms of practical implications such as product cost, food safety and ease of production in developing countries, which are important issues in treatment of SAM. By using milk-free RUTF, the cost of the final product may be reduced by approximately 10 to 25% compared to the cost of PM-RUTF based on differences in raw materials cost because skim milk is expensive compared to grains. Furthermore, since milk protein relies on imports from developed countries, plant-based RUTFs will rely more on locally available ingredients, ultimately reducing the logistical costs and boosting local labor creation. In addition, removing peanut from the ingredients will eliminate concerns regarding peanut allergies and the high risk of aflatoxin contamination of the product. This product may be a good alternative for the treatment of not only children but also malnourished adults with lactose intolerance. 65–75% of adults worldwide are lactose intolerant [35]; in malnourished adults secondary temporary lactose intolerance is likely highly prevalent.

In conclusion, the plasma EAA concentrations following FSMS-RUTF and MSMS-RUTF treatments were not less than those following PM-RUTF treatment in 6-59-mo-old children who were diagnosed with SAM. We believe that the requirements for adequate protein synthesis and a sufficient supply of functional metabolites of amino acids such as GSH were met and contributed to the SAM recovery rates observed in all arms [5]. This might contribute to cost reduction for therapeutic products and to the ability to treat adults and children with lactose intolerance and peanut allergies.

## Supporting information

S1 CONSORT Checklist CONSORT checklist for the trial. (DOC)

S1 Table Nutritional composition of the study RUTFs*. ^1^FSMS = Milk-free soy-, maize-, and sorghum-based ready-to-use therapeutic food; ^2^MSMS = Milk-, soy-, maize-, and sorghum-based ready-to-use therapeutic food; ^3^PM = Peanut paste-based ready-to-use therapeutic food.
^4^RE, retinol equivalent; TE, total energy; SFs, saturated fatty acids; MUFAs, monounsaturated fatty acids; PUFAs, polyunsaturated fatty acids.
*This table is reused from our primary outcome paper (Bahwere P et. al., AJCN, 2017).
(DOCX)

S2 Table Comparison of the amino acid profiles of the studied RUTFs obtained by laboratory analysis*. ^1^FSMS = Milk-free soy-, maize-, and sorghum-based ready-to-use therapeutic food; ^2^MSMS = Milk-, soy-, maize-, and sorghum-based ready-to-use therapeutic food; ^3^PM = Peanut paste-based ready-to-use therapeutic food; ^4^Glu+Gln = Glutamic acid or glutamine; ^5^Asp+Asn = Aspartic acid or asparagine
*This table is reused from our primary outcome paper (Bahwere P et. al., AJCN, 2017).
(DOCX)

S3 Table Testing noninferiority of the plasma amino acid concentrations at discharge between the FSMS-RUTF arm and the MSMS-RUTF and PM-RUTF arms in the age subgroup^1^. ^1^The point estimate and 95% CI of difference in plasma amino acid concentrations at discharge between the FSMS-RUTF arm and PM-RUTF arm and between the MSMS-RUTF arm and PM-RUTF arm by using the age subgroup data.
^2^CIs were estimated by simultaneous inference procedures in mixed model.
^3^Noninferiority margins were -25% of the plasma amino acid concentrations of the PM-RUTF arm at discharge.
FSMS, milk-free soy, maize, and sorghum; MSMS, milk, soy, maize, and sorghum; PM, peanut and milk; RUTF, ready-to-use therapeutic food; CI, confidence interval; NI, noninferiority; BCAA, branched-chain amino acid; EAA, essential amino acid.
(DOCX)

S1 Study Protocol Study protocol of acceptability and efficacy of an innovative soya based RUTF for the treatment of severe acute malnutrition. (PDF)

S1 Dataset Dataset of plasma amino acid concentrations in study participants. (XLSX)
