# Supplementary material for: Amino-acid-enriched cereals ready-to-use therapeutic foods (RUTF) are as effective as milk-based RUTF in recovering essential amino acid during the treatment of severe acute malnutrition in children: An individually randomized control trial in Malawi
Source: PLoS One. 2018 Aug 10;13(8):e0201686. doi: 10.1371/journal.pone.0201686 (PMC6086422; doi:10.1371/journal.pone.0201686)
Supplement: S1 Table — 1FSMS = Milk-free soy-, maize-, and sorghum-based ready-to-use therapeutic food; 2MSMS = Milk-, soy-, maize-, and sorghum-based ready-to-use therapeutic food; 3PM = Peanut paste-based ready-to-use therapeutic food. 4RE, retinol equivalent; TE, total energy; SFs, saturated fatty acids; MUFAs, monounsaturated fatty acids; PUFAs, polyunsaturated fatty acids. *This table is reused from our primary outcome paper (Bahwere P et. al., AJCN, 2017). (DOCX) [file pone.0201686.s002.docx]

**S1 Table.** **Nutritional composition of the study RUTFs**^*^

^1^FSMS=Milk-free soy-, maize-, and sorghum-based ready-to-use therapeutic food; ^2^MSMS=Milk-, soy-, maize-, and sorghum-based ready-to-use therapeutic food; ^3^PM=Peanut paste-based ready-to-use therapeutic food.

^4^RE, retinol equivalent; TE, total energy; SFs, saturated fatty acids; MUFAs, monounsaturated fatty acids; PUFAs, polyunsaturated fatty acids.

*This table is reused from our primary outcome paper (Bahwere P et. al., AJCN, 2017).
