# Supplementary material for: Amino-acid-enriched cereals ready-to-use therapeutic foods (RUTF) are as effective as milk-based RUTF in recovering essential amino acid during the treatment of severe acute malnutrition in children: An individually randomized control trial in Malawi
Source: PLoS One. 2018 Aug 10;13(8):e0201686. doi: 10.1371/journal.pone.0201686 (PMC6086422; doi:10.1371/journal.pone.0201686)
Supplement: S2 Table — 1FSMS = Milk-free soy-, maize-, and sorghum-based ready-to-use therapeutic food; 2MSMS = Milk-, soy-, maize-, and sorghum-based ready-to-use therapeutic food; 3PM = Peanut paste-based ready-to-use therapeutic food; 4Glu+Gln = Glutamic acid or glutamine; 5Asp+Asn = Aspartic acid or asparagine *This table is reused from our primary outcome paper (Bahwere P et. al., AJCN, 2017). (DOCX) [file pone.0201686.s003.docx]

**S2 Table.** **Comparison of the amino acid profiles of the study RUTFs obtained by laboratory analysis**^*^

^1^FSMS=Milk-free soy-, maize-, and sorghum-based ready-to-use therapeutic food; ^2^MSMS= Milk-, soy-, maize-, and sorghum-based ready-to-use therapeutic food; ^3^PM= Peanut paste-based ready-to-use therapeutic food; ^4^Glu+Gln=Glutamic acid or glutamine; ^5^Asp+Asn=Aspartic acid or asparagine

^*^This table is reused from our primary outcome paper (Bahwere P et. al., AJCN, 2017).
