# Supplementary material for: Amino-acid-enriched cereals ready-to-use therapeutic foods (RUTF) are as effective as milk-based RUTF in recovering essential amino acid during the treatment of severe acute malnutrition in children: An individually randomized control trial in Malawi
Source: PLoS One. 2018 Aug 10;13(8):e0201686. doi: 10.1371/journal.pone.0201686 (PMC6086422; doi:10.1371/journal.pone.0201686)
Supplement: S3 Table — 1The point estimate and 95% CI of difference in plasma amino acid concentrations at discharge between the FSMS-RUTF arm and PM-RUTF arm and between the MSMS-RUTF arm and PM-RUTF arm by using the age subgroup data. 2CIs were estimated by simultaneous inference procedures in mixed model. 3Noninferiority margins were -25% of the plasma amino acid concentrations of the PM-RUTF arm at discharge. FSMS, milk-free soy, maize, and sorghum; MSMS, milk, soy, maize, and sorghum; PM, peanut and milk; RUTF, ready-to-use therapeutic food; CI, confidence interval; NI, noninferiority; BCAA, branched-chain amino acid; EAA, essential amino acid. (DOCX) [file pone.0201686.s004.docx]

**S3 Table.** **Testing noninferiority of the plasma amino acid concentrations at discharge between the FSMS-RUTF arm and the MSMS-RUTF and PM-RUTF arms in the age subgroup^1^**

^1^The point estimate and 95% CI of difference in plasma amino acid concentrations at discharge between the FSMS-RUTF arm and PM-RUTF arm and between the MSMS-RUTF arm and PM-RUTF arm by using the age subgroup data.

^2^CIs were estimated by simultaneous inference procedures in mixed model.

^3^Noninferiority margins were -25% of the plasma amino acid concentrations of the PM-RUTF arm at discharge.

FSMS, milk-free soy, maize, and sorghum; MSMS, milk, soy, maize, and sorghum; PM, peanut and milk; RUTF, ready-to-use therapeutic food; CI, confidence interval; NI, noninferiority; BCAA, branched-chain amino acid; EAA, essential amino acid.
