# Supplementary material for: Amino-acid-enriched cereals ready-to-use therapeutic foods (RUTF) are as effective as milk-based RUTF in recovering essential amino acid during the treatment of severe acute malnutrition in children: An individually randomized control trial in Malawi
Source: PLoS One. 2018 Aug 10;13(8):e0201686. doi: 10.1371/journal.pone.0201686 (PMC6086422; doi:10.1371/journal.pone.0201686)
Supplement: S1 Study Protocol — (PDF) [file pone.0201686.s005.pdf]

Study Protocol

Acceptability and Efficacy of an Innovative Soya Maize Based RUTF for the Treatment of Severe Acute Malnutrition

29 October 2014

Valid Nutrition for a partnership study with Ajinomoto

1 Executive summary

1.1 Background:

Ready to Use Therapeutic Foods (RUTF) have been used widely in Community based Management of Acute Malnutrition programmes (CMAM) to treat severe acute malnutrition (SAM) in children in resource-poor settings. Hitherto, most of the RUTF used in CMAM programmes uses cow milk as the main protein source. The use of cow milk at 25% by weight of the final product in these formulations makes them too expensive for sustainable use in resource-poor settings. At the same time attempt to produce free or low milk content RUTFs has yielded contrasting results with the most promising formulation being as effective as the standard formulation only in children aged 24 months or more. Thus, innovative free or low cow milk alternatives are needed.

Only two studies both conducted by Valid, have tested alternative milk-free recipes that are based on locally produced cereals and legumes for efficacy among SAM children. These studies have revealed that this milk-free RUTF is as effective as the standard RUTF in children two years and more but less effective than the standard RUTF in children less than two years at the beginning of treatment. Possible explanations for this include sub-optimal levels of key amino acids (AA) involved in protein synthesis during this period of rapid growth. Studies have recently demonstrated that SAM children have high requirements of certain AA and that supplementation with these AA at the initiation of the treatment or during the phase of catch up growth may improve protein synthesis and support faster recovery.

One of Valid's studies included a component of body composition assessment using the deuterium dilution technique in one sub-sample and using bio-electrical impedance in another sub-sample. The deuterium revealed that none of RUTF was associated with excess fat deposition. Both sub-studies revealed that they may be a subtle difference between the two RUTFs that could partially explain the quality of synthesised tissues. From the results it could be hypothesised that catch up in linear growth may happen at the expense of repair of cellular membranes. These findings need to be confirmed in a study with a bigger sample size and subsequently the AA profile of the new SMS-RUTF should be improved to be near equivalent to that of the reference protein.

The international nutrition community are now pushing for an integration of programs that address stunting with programs that address acute malnutrition. Data have shown that most children with severe acute malnutrition are also stunted. Having a product that will reverse wasting while also promoting catch up in linear growth is needed. Such a product will support feasibility and success of the process of integration.

1.2 Goal:

The goal of this research programme is to assess in two different phases the acceptability (phase 1) and the efficacy (phase 2) of two innovative Soya-Maize-Sorghum RUTFs for the treatment of SAM in children under five years old. The two products are the new version of the Milk Free Soya-Maize RUTF (FSMS-RUTF) and the 10% Milk Soya-Maize-Sorghum RUTF (MSMS-RUTF). The new SMS-RUTF has an improved AA profile compared to the previous milk free SMS-RUTF and less phytic acid.

Phase 1: acceptability
Primary hypotheses:

1. FSMS-RUTF and MSMS-RUTF have organoleptic characteristics (taste, smell, texture, colour) as acceptable as the standard milk and peanut based RUTF (P-RUTF) for Malawian children aged 3 to 5 years attending community based child care centres (CBCC)

2. FSMS-RUTF or MSMS-RUTF intake will not be associated with more RUTF related side effects than P-RUTF in Malawian children aged 3 to 5 years attending CBCC.

Secondary hypothesis:

3. FMSMS-RUTF and MSMS-RUTF will not be associated with a higher level of breath hydrogen excretion when compared to P-RUTF.

Phase 2: efficacy
Primary hypotheses:

1. Recovery rate of children aged 6 to 23 months with SAM treated with FSMS-RUTF is > 75% and is not inferior to that of children with SAM of the same age treated with MSMS-RUTF nor to that of P-RUTF with the fixed non-inferiority margin being 10%.

2. Recovery rate of children aged 24 to 59 months with SAM treated with FSMS-RUTF is > 75% and is not inferior to that of children with SAM of the same age treated with MSMS-RUTF nor to that of P-RUTF with the fixed non-inferiority margin being 10%.

Principal secondary hypotheses:

1. Weight gain (g/kg/day) during of children aged 6 to 59 months with SAM treated with FSMS-RUTF is not inferior to that of children with SAM of same age treated with MSMS-RUTF nor to that of those treated with P-RUTF with the fixed non-inferiority margin being 1.5g/kg/day (age group 6 to 23 months and 24 to 59 months to be sampled and analysed separately).

2. Time to recovery under treatment from admission to recovery of children aged 6 to 59 months with SAM treated with FSMS-RUTF is within the non-inferiority margin of 14 days when compared to children of the same age treated with MSMS-RUTF or to P-RUTF (age group 6 to 23 months and 24 to 59 months to be sampled and analysed separately).

3. Height gain during recovery and 3 months after discharge of children aged 6 to 59 months (admission age) with SAM treated with FSMS-RUTF is not inferior to that of children with SAM of the same age treated with MSMS-RUTF nor to that of children treated with P-RUTF given a fixed non-inferiority margin of 0.5 cm (age group 6 to 23 months and 24 to 59 months to be sampled and analysed separately).

4. Proportionally to body weight, percentage of body fat mass (BFM) measured at the time of recovery of children treated with FSMS-RUTF will not be higher than the percentage of BFM in children treated with MSMS-RUTF nor higher than the percentage of BFM of those treated with P-RUTF at the fixed margin of inferiority of 6%.

5. Tissues electrical properties assessed by Bio-electrical impedance analysis (BIA) will not be impaired in children treated with FSMS-RUTF when compared to that of children treated with MSMS-RUTF or with P-RUTF given a margin of equivalence to be determined by the synthesis methods based on findings of the SMS-RUTF study recently completed in DRC.

Other secondary hypotheses:

6 Haemoglobin gain of children treated for SAM with FSMS-RUTF will not be lower than that of children treated with MSMS-RUTF nor than that of those treated with P-RUTF given a margin of non-inferiority of 0.5g/dl.

7 The RUTF daily intake in grams and in kcal/kg/day of children 6 to 59 months old treated for SAM with FSMS-RUTF will not be inferior to the daily intake of children of the same age treated with MSMS-RUTF nor to the daily intake of those treated with P-RUTF given a margin of non-inferiority of 18g/day and 25kcal/kg/day (age group 6 to 23 months and 24 to 59 months to be sampled and analysed separately)

8 Breast milk intake of children 6 to 23 months old treated for SAM with FSMS-RUTF will not be lower than the breast milk intake of children of the same age treated with MSMS-RUTF nor to the intake of those treated with P-RUTF given a margin of non-inferiority of 100g/day.

9 Proportion of children with normalised stool calprotectin at 14 days, 28 days and recovery will not be lower in children treated for SAM with FSMS-RUTF than in those treated with MSMS-RUTF or in those treated with P-RUTF given a margin of non-inferiority determined using the synthesis method based on the confidence interval observed in children treated with P-RUTF.

10 Plasmatic level of key growth related AA of children treated with FSMS-RUTF will not be inferior to that of children treated with MSMS-RUTF or P-RUTF given a margin of non-inferiority defined using the synthesis method

11 12-months after discharge survival in children with SAM treated with FSMS-RUTF will not be inferior to that of children treated with MSMS-RUTF nor to that of those treated with P-RUTF given a margin of non-inferiority of 10%.

1.3 Rationale:

P-RUTF has been shown to be efficacious in the treatment of SAM in children but it contains 25% cow milk-powder that is often exclusively imported. This makes local production in affected developing countries complex and costly. The use of alternative foodstuffs that are locally grown may offer an affordable option. Further, the utilisation of locally grown foodstuffs for RUTF production may provide ready markets for agricultural produce, and in this way, increase the income of local people. Available data confirm that milk-free RUTF can be use as alternative to the standard P-RUTF in children 24 months or older but that there is need of optimisation of the formulation for those below 24 months of age. The present study will test a modified formulation of SMS-RUTF. The modification in the formulation consists of improving the amino acid profile using an amino acid mixture (FSMS-RUTF) or including cow milk at 10% weight of the final product. It is expected that these changes will improve recovery rate in children of all ages.

1.4 Potential impact on child health:

There is good evidence of the benefit of RUTF in reducing case-fatalities among children suffering from SAM. Its use in CMAM programming has been shown to reduce SAM related mortality from 20-30% to below 5% while increasing programme coverage from below 10% to around 50%. It has also been shown that health benefits may extend to achieving better long term survival than the general population of the same age and same community. Emerging evidence suggest that there may be relationship between severe acute malnutrition and stunting. It is likely that improving the coverage of SAM treatment may also contribute to the reduction of the burden of stunting.

1.5 Methods:

1.5.1 Design*:*

The acceptability trial will be a cross-over study with the two RUTFs to be tested compared individually with the standard RUTF. At this stage there will not be comparison between FSMS-RUTF and MSMS-RUTF. The

efficacy trial will be a three arms statistician blinded, randomized and controlled, parallel group, efficacy non-inferiority trial. The two study foods will be compared individually with the standard RUTF and there will also be a comparison between the two study foods.

1.5.2 Methodological approaches and procedures:

1.5.2.1 Acceptability trial

Children aged 3 to 5 years with MUAC below 12.5 cm regularly attending the selected community based child care centres (CBCC) will be eligible for the acceptability trial. Children will be divided in 4 groups. In 2 separate phases, each group will alternatively receive one of the study food (FSMS-RUTF or MSMS-RUTF) and the control food (P-RUTF). Two CBCC will be used with each site testing a separate study food. Three meals per day of either the study food or the control food will be served 5 days per week during 2 consecutive weeks at the dose of 92g/day. The 2 phases will be separated by two weeks (washout time).

MUAC, weight and height measurements, body temperature, respiratory rate, a clinical assessment when required, hydrogen breath test, observation of general response to food administered and a caregiver's interview will be the only procedures carried out during the acceptability trial. Data will be collected daily, on pre-defined data collection form. Hired research enumerators will collect the data with the help of CBCC workers.

1.5.2.2 Efficacy trial

Children staying within 1 hour walking distance from feeding points especially established in the community for the study will be enrolled and will be followed up from admission to nutrition recovery using a day care approach. Enrolled children will attend the site daily from 9 am to 3 pm. Children will be offered 200kcal/kg/day of one of the study RUTF. Children will be declared non-recovered if they don't meet the discharge criteria (MUAC≥ 12.5 cm and no oedema) after 3 consecutive months of treatment. Apart from the difference in study therapeutic food, children of all the three study groups will be treated in the same way in accordance with the national guidelines for the treatment of SAM but using the day-care approach as stated above. The day-care approach allows the elimination of several confounding factors such us sharing or poor intake of the therapeutic food at households' level, inappropriate intake of routine medicines and differences in the product acceptance by caregivers that may affect child feeding of the study food. This approach will also allow close monitoring of side effects such us abdominal pain due to iron intake, flatulence (due to unabsorbed sugar), diarrhoea due to lactose intolerance and appetite suppression due to some oligosaccharides. Anthropometric parameters will be measured daily (weight and MUAC) or weekly (height). Clinical conditions will be assesses daily (appetite, oedema, cough, difficulty of breathing, diarrhoea, dehydration, fever, skin eruption, neurological status, anaemia). Embedded sub-studies will allow the assessment of

- haemoglobin (admission and discharge) with an Haemocue device that uses capillary blood collected at finger prick;
- body composition using deuterium dilution technique (at discharge for both those who recovered and those who did not recover);
- body composition using bio-electrical impedance analysis (at admission or when oedema subside, at time of reaching discharge criteria, at discharge and at 3 months follow up) using a Bodystat 1500 MDD device;
- breast milk intake using the dose-to-mother deuterium oxide dilution technique in children still breastfed at admission (two weeks after the start of the treatment);
- stool calprotectin using a quantitative point-of–care chromatographic immunoassay kit for the assessment of intestinal inflammation (admission, 28 days after the start of treatment, at discharge)
- free serum amino acid profile using venous blood.
- Post-discharge mortality will be assessed 12 months after nutrition recovery.

All data will be collected on a modified version of the standard clinic cards used for monitoring treatment of SAM. Data of some specific procedures including body composition assessment, breast milk intake assessment and amino acid profile assessment will be collected on separate forms specially designed for the sub-studies.

1.5.3 Sample size:

1.5.3.1 Acceptability trial

We will use a convenience sample of 20 children per group (40 children per study site) equivalent to 40 children testing one study food in a cross-over design and a total of 80 children used for the acceptability trial (2 sites). At each site 20 children will start with a study food (FSMS-RUTF or MSMS-RUTF) and shift to P-RUTF during phase 2 of acceptability. Twenty other will start with P-RUTF and shift to the study food during phase 2 of acceptability.

1.5.3.2 Efficacy trial

The sample size is calculated based on the primary objectives only. A minimum of 750 SAM children below 24 months of age and 450 SAM children 24 to 59 months of age is required to be 80% sure that the upper limit of a one-sided 95% confidence interval will exclude a difference in recovery rate in favour of the standard group of more than 10%. In addition 60 community control children will be enrolled for the Deuterium dilution technique sub-study. The number of children to be enrolled as community control of children discharged alive and for whom the follow up is possible will depend on the number of children meeting follow up eligibility criteria.

1.5.4 Analysis:

1.5.4.1 Acceptability trial

The acceptability and the tolerance will be judged based on the criteria listed below

- Persistence of good appetite
- Consumption of more than 75% of the offered food within 1 hour. (meal acceptance)
- Consumption of more than 75% of the offered food for more than 75% of the days on trial (overall acceptance)
- Frequency of ill-effects associated with the food requiring withdrawal from the trial is less than 10%.
- Consumption of a quantity of energy not less than 75% of the energy intake from the control food.

The organoleptic characteristics (taste, colour, smell, texture) will be assessed using a 5-points likert scale validated for the age group 3 to 5 years. Data will be analysed by comparing the mean scores of the study foods (FSMS-RUTF or MSMS-RUTF) for each organoleptic characteristic with the score of the control food which is the P-RUTF.

1.5.4.2 Efficacy trial

Appropriate quantitative analysis will be used including descriptive statistics (proportion, percentage, mean or median), comparison of difference in proportion for binary outcome and comparison of average (mean or median as appropriate) change for continuous variable. Both intention to treat (ITT) and per protocol (PP) approaches will be used for data analysis and reporting. Data of children below 24 months and that of those aged 24 months or more will be analysed separately except for body composition and haemoglobin. Subgroup analyses assessing the effect of the intervention in different subgroups will be carried out. The variables considered for the sub-group analyses include gender, type of malnutrition (oedematous and non-oedematous) stunting and HIV status (positive or negative). Interactions between the interventions and other variables will be checked and data analysis scheme adjusted accordingly in case of a statistically significant interaction. No interim analysis will be performed except if the monitoring and safety committee identify need for such analysis.

1.6 Study sites:

The study will be carried out in Malawi. Three Health District of Malawi have been recommended by the Ministry of Health including Lilongwe, Dedza and Mchinji. In these Health Districts we will select the Clusters (a sub-administrative area in Malawi administrative organisation) with the highest burden of acute malnutrition according to the monthly routine statistics. In total, 30 community based feeding centres will be established (10 in each District) in the catchment areas of OTP sites that reported more than 60 admissions of SAM cases in 2013.

1.7 Ethical consideration:

The study will be conducted in compliance with the Declaration of Helsinki. Permission for the trial to be conducted will be obtained from the National Health Sciences Research Committee and the Ajinomoto institutional review board. Participation will be voluntary. At the time of admission, each child's parent or carer will be informed about the nature and purpose of the study and asked for their verbal and written consent for their child to be included in the study and for their medical information to be used for research purposes. The carer information sheet and the consent form will be in English but will be also translated in Chichewa, the national language. The sheet will contain information on the study objectives, study procedures and anticipated duration of participation, potential risks and discomfort for the child, benefits for the child and for the under-five population in general, condition of study termination or participant withdrawal, confidentiality of records and contact persons in case any question or problem arise. When parents or carer withhold consent for participation, children will be referred to non-participating clinics providing the standard of care for SAM children. There are several such clinics in the selected Districts. They use standard P-RUTF. Any child who develops study food related side effects or any serious disease during the study will be referred to the appropriate level of care and the cost of the treatment met by the research project. Participating children and all SAM children will benefit from study. A committee of experts will be appointed to serve as the data monitoring and safety committee. They will be notified monthly of the trial's progress and of all the cases of study related side effects as they are diagnosed.

1.8 Study Team:

Valid: Paluku Bahwere, M.D, PhD (Principal Investigator), Kate Sadler, PhD., Peter Akomo, Msc., Theresa Banda, Msc, Bsc., and Steve Collins, M.D., PhD.
Ajinomoto CO., INC._: Hitoshi Murakami, PhD.
Free University of Brussels: Paluku Bahwere, M.D, PhD., Michèle Dramaix-Wilmet, PhD.
Ministry of Health Malawi: Sylvester Kathumba, Msc., Mwayi Mwale, M.D., Salomon Jere, M.D., Chimwemwe Banda M.D.

2 Detailed description of the study

2.1 Literature review:

Severe Acute Malnutrition (SAM) affects approximately 19 million children under the age of 5 and is associated with between one and two million preventable child deaths each year [1, 2]. In most developing countries, Case Fatality Rates (CFRs) in hospitals treating SAM remain at 20-30% many years after the standardised WHO protocol has became available and few of those requiring care actually access treatment [2-4]. SAM is defined as a weight-for-height measurement of 70% or less below the median, or three Standard Deviations (SD) or more below the new WHO growth standard, the presence of bilateral pitting oedema of nutritional origin, or a Mid-Upper-Arm Circumference (MUAC) of less than 11.5 cm in under five children [5]. In community based programme, the definition of SAM is based mainly on MUAC and presence of bilateral pitting oedema as described above[5].

Community-based management of SAM (CMAM) has been developed to offer a new approach to delivering care to acutely malnourished children in emergency situations and in more stable settings[6, 7]. The model is rooted in public health principles of coverage and access and is designed to achieve population-wide impact [8]. It focuses primarily on treatment of the majority of the acutely malnourished people as outpatients in their homes rather than in Therapeutic Feeding Centres (TFCs)[8]. Intensive inpatient care is also provided for those who have complications[8]. Techniques of community mobilisation are used to engage the affected population and achieve high proportion of early presentation while maximising coverage[8].

A recent United Nations (UN) joint publication summarizing experience with the community-based management of SAM concluded that CFRs of lower than 5% can be achieved through community-based management of SAM [5, 9]. CFR among 23,511 unselected severely malnourished children treated in 21 CTC programmes in Malawi, Ethiopia, and Sudan was 4.1%; with recovery rate and default rate of 79.4% and 11.0% respectively [9, 10]. Several recent studies have demonstrated the effectiveness of the CMAM in Malawi [11-16] and in other countries in Africa and Asia[17-22].

It has been possible to implement CMAM programmes for children with SAM only since the development of Ready-to Use Therapeutic Food (RUTF) [23-25] RUTF is a lipid-based paste that is energy dense, resists bacterial contamination, and requires no cooking[24, 25]. The primary production principles include grinding all ingredients to particle size of < 200 microns, producing the food without the introduction of water, and embedding the protein and carbohydrate components of the food into the lipid matrix [24-26]. The water free nature makes RUTF resistant to bacterial contamination[25]. The most widely used RUTF is a mixture of milk powder, sugar, vegetable oil, peanut butter, vitamins, and minerals [24-26]. It is equivalent to the WHO F-100 milk. RUTF have been used widely in CTC to treat severely malnourished children in resource-poor settings and the global volume of sell has been increasing because of the momentum created by CMAM and because to date the proportion of caseload that access treatment yearly remain very low but is on the increase [27-29]. High recovery rates, lower case-fatalities, and greater weight gain have been demonstrated using RUTF [2, 7, 30-32]. A study conducted in Malawi on children with SAM treated using RUTF has also demonstrated that the majority (over 85%) of children discharged as cured from a programme using the CMAM approach maintained a normal weight for height for as long as 15 months after discharge [11].

To date, there has been little success in the expansion of local production of RUTF. Most of the RUTFs used in programmes is still produced in industrialised countries mainly Europe[29]. Several reasons explain this situation including difficulties of procuring quality ingredients in Africa and the cost of importing the key ingredients. The problematic ingredients in the P-RUTF are the peanut paste and cow's milk powder. Peanut paste is difficult to produce for local farmers and food processing companies because of the problem of aflatoxin contamination. A recent study has for example revealed that most of the processed complementary and therapeutic foods that are available in Malawi have unacceptable levels of aflatoxins[33]. This situation

suggests that an effective peanut paste free formulation is needed. Valid has tested alternative formulations such us Chickpea and Sesame based RUTF. They were effective in reversing wasting in adults but experience of their use in children is limited [16, 34].

The intensive use of milk in the formulation of the current standard RUTF makes it too expensive for sustainable use in resource-poor settings. Valid Nutrition has developed alternative milk-free recipes based on locally produced foodstuffs including the SMS-RUTF. Two randomised studies one already published and one just completed have assessed the efficacy of the SMS-RUTF and have demonstrated that it is as effective as P-RUTF in treating SAM in children 24 months or older[35]. In children less than 24 months although over 50% respond well, the proportion of non-respondent was significantly higher than among children treated with P-RUTF [35]. This finding is very important as it shows that the most expensive P-RUTF could be restricted to younger children to minimise the cost of the programme. However, in terms of programme logistic it might be very difficult to handle two different products and the optimisation of the SMS-RUTF to improve its efficacy in children less than 24 months is highly desirable. This means more research to understand the physiology of children less than two years and to identify the reasons for poor response to the milk-free RUTF in children less than 2 years is needed. For this group breast-milk could theoretically complement the RUTF and improve the intake of essential nutrients including essential amino acids. Although, several studies have shown that lipid based paste used for preventing or treating malnutrition does not affect breast milk intake of infant, it will be necessary to confirm that this is still the case when infants are prescribed as much as 200kcal/kg/day[36-38].

Although possible, it is unlikely that the lower recovery rate among under-twos was due to higher content of phytic acid because a deliberate effort to reduce phytates was made during processing and because P-RUTF also has high levels of phytic acid. Indeed, a recent laboratory analysis of different P-RUTFs used in humanitarian programming found that even the P-RUTF produced in Europe is far from reaching the acceptable phytate/iron molar ratio of <1 because it has a high concentration of phytates (1015mg/100g) and a low concentration of iron (10.41 mg/100g) [39]. The SMS-RUTF used in our DRC SMS-RUTF study had a phytate content of 420 mg/100g that is closer to the level of 371mg/100g in the sample of P-RUTF obtained in an African factory by the author of the paper[39]. The effort to improve the phytates/iron molar ratio was complemented by an increase in iron and zinc content to improve the phytates/zinc and phytates/iron molar ratios and an increase in Vitamin C content to improve iron absorption. These strategies have been found to improve absorption of these minerals especially of iron [40, 41].

Concerns remain regarding the level of iron in therapeutic or supplementary food despite some studies showing the positive effect of iron supplementation on growth [42]. These concerns are related to the potential side effects that some studies have attributed to iron fortified food of inducing an increase in the population of pathogenic bacteria in the gut[43, 44]. In the case of RUTF, and especially of SMS-RUTF that may have higher level of phytates than P-RUTF, an increase in iron content was justified by the fact that an unpublished study conducted in Senegal showed that treatment with P-RUTF was not associated with a significant increase in haemoglobin (HB) while treatment with the therapeutic milk (F100) fortified with iron to provide 3mg/kg/day was associated with a significant increase in haemoglobin [45]. In the F100 group, HB increased from 83.1 g/L (95% CI: 77.0-89.1) to 91.4 g/L (95% CI: 86.1-96.7) during rehabilitation ($P < 0.001$). In the RUTF group, HB increased from 85.4 g/L (95% CI: 80.4-90.3) to 87.1 g/L (95% CI: 80.9-93.2)[45]. The associated iron deficiency anaemia among children with SAM was confirmed in another recent study conducted in Turkey[46]. Also, a recent study in Malawi has suggested that iron can be safely prescribed to children recovering from severe malaria, a condition that in the past has been associated with very high post-discharge mortality [47, 48].

Despite several studies that have shown that nutrition therapy with appropriate diet is associated with increase in free fat lean mass including muscle volume increase, there has been a continued debate around the possible association between rapid weight catch up growth observed during nutrition rehabilitation of SAM, higher amounts of body fat deposition and insufficient repletion of muscle and visceral proteins – all occurring without full corrections of vitamins and mineral deficiencies[49-52]. Data from the recently completed SMS-RUTF

study in DRC has shown that the fear of excess fat deposition is not justified when RUTF is used as there was no excess fat deposition either with SMS-RUTF or with P-RUTF when compared to community controls. In addition, the Bio-electrical impedance (BIA) parameters collected during this study were still improving at the time of discharge, suggesting that the recovery of the electrical tissues properties was not complete.

Some experts believe that height gain is the best anthropometric indicator of increase in lean mass during nutrition rehabilitation and that weight gain without linear catch up may just indicate an increase in body fat[53]. However, the duration of nutrition rehabilitation for SAM is too short to demonstrate height catch up as many studies have demonstrated that weight gain precedes height gain by 3 to 4 months [49, 52, 54-56]. In addition it seems that height increment does not begin until children recovering from SAM have achieved at least 85% of expected weight-for-length meaning that they are usually discharged from therapeutic feeding program before height increment is seen [52]. Indeed, studies that have reported height gain during nutrition rehabilitation reported a modest height gain rate of around 0.2 mm/day [12]. Thus, height gain should be rechecked 3 to 6 months after recovery to identify any impact of treatment of SAM on catch up of linear growth. A study that distributed P-RUTF to prevent acute malnutrition during a hunger season in Niger has suggested that such catch up may occur [57].

A study in Uganda has revealed that fatty acid metabolism is important in the development of and recovery from SAM[58]. The findings of the study suggest that fatty acid metabolism regulate the adaptation to SAM with low levels of the adipose tissue hormone leptin being a strong predictor of the severity of and death from SAM[59]. The study also showed that plasmatic levels of many amino acids is reduced at the start of treatment suggesting contribution of protein metabolism to the phenomenon[58]. The serum level of the AA tested increased to maximum level within two weeks[58]. During that period, children were on the milk diet. There is no published similar data with RUTF especially for milk free RUTF. The lack of that information complicates optimisation of free milk RUTFs.

Authors have incriminated the formerly called tropical enteropathy and now called environmental enteric dysfunction (EED) in the development of malnutrition and in the response to its treatment [60]. EED is believed to result from very high concentrations of ingested faecal bacteria in the small-intestinal lumen and subsequent unrestrained enteric T-cell activation [61]. EED affecting almost every child in developing countries is characterised by villous atrophy, crypt hyperplasia, increased permeability, inflammatory cell infiltrate and malabsorption [62]. Authors believe that it can explain up to 39% and 43% of weight and linear growth respectively [62, 63]. These findings suggest that interventions focusing on gut microbial populations might be as important as those focusing on improving infant diets in the fight against acute and chronic malnutrition [64, 65]. Indeed, a study conducted in Malawi on twin pairs has implicated gut microbiome in the aetiology of kwashiorkor by showing that gut microbiome of children with Kwashiorkor induce weight loss in in germ-free mice and that kwashiorkor is associated with presence of reduced concentration of" beneficial flora" and high concentration of "non-beneficial flora"[66]. The study also revealed that treatment with standard RUTF only transiently improves gut microbiome[66]. This has been confirmed in another study conducted in Bangladesh that has shown that with current therapeutic approaches and foods used, immaturity of gut microbiome persists after SAM children have been declared cured based on anthropometry [67]. These two studies suggest that new approaches of treatment should prove both replenishment of nutrients and muscles and restoration of gut normality including normal flora and normal mucosal histology and function [60, 66, 67]. This can be demonstrated by examining stools for microbiome population and determining concentration of calprotectin in stools as markers of intestinal mucosa inflammation[60]. While it is logistically difficult to organise samples storage for microbiome analysis as it require storage at -70 °C and sample analysis that required sending samples abroad, it is possible in community based programmes to measure level of calprotectin in the stools as point of care tests have been made available[68]. Also, it has been shown that calprotectin can be an indirect indicator of gut microbiome characteristics[69].

Although the information available on the cost-effectiveness of therapeutic feeding centres, and especially community-based management approaches, is sufficient to justify the inclusion of the management of SAM in the child survival strategy, there is still resistance from some policy makers and donors to invest in this intervention over the long term [10, 18, 70-74]. A commonly cited reason behind this resistance is the supposed persistence of a high risk of mortality after recovery from SAM once the patient has exited a treatment programme [75-78]. Data on the longer term mortality associated with discharge from inpatient therapeutic feeding centres is variable; some studies have reported a cumulative mortality rate after 1 to 1.5 years from discharge of up to 41% [75, 76, 79-82], whilst others have reported lower figures of 2.3% after 12 months of follow up, and 4.1% after 1.5 years of follow up [11, 83]. An earlier study reported a mortality of 1.5%, 12 months after discharge among children treated using the domiciliary approach [83]. None of these studies assess the reasons behind the high mortality after discharge from therapeutic feeding programmes, nor do they examine how post-discharge mortality compares with the mortality rate of all children in the same community. Given the strong association between high childhood mortality, poverty and SAM, it may be that baseline mortality rates partly explain both the observed high post discharge mortality and the study- to-study variation. One study that reviewed the existing evidence on long term mortality has shown that children treated in CMAM programmes who start nutrition rehabilitation at an early stage of metabolic malnutrition have long term survival that is comparable to that of children in the same community [84].

2.2 Background and rationale

As indicated above, acute malnutrition remains a public health problem in many developing countries including Malawi and its burden is currently underestimated. This is because current estimates are based on prevalence and not on the actual incidence of the condition and because the caseload generated by natural and man-made disasters are not included[28]. In Malawi, acute malnutrition affects 4% of all under-five children and across the country thousands of children are treated for SAM each year using the CMAM approach.

Children with SAM need safe, palatable foods with energy, protein, fat, minerals and vitamins tailored to their needs for restoration of normal body functions and catch up growth [5]. Providing P-RUTF tailored to body weight has been shown to successfully support catch-up growth [2, 10, 32]. But, P-RUTF is expensive and the cost affects the coverage and the sustainability of CMAM programmes. Almost half of the cost of the P-RUTF is due to milk powder that constitutes 25 to 30% of the content of P-RUTF. Hence at the moment, there is a dire need to produce and assess the efficacy of a milk free RUTF.

Although it is understood that peanut allergy is not common in resource poor countries, and that it may not be a major concern for programs promoting formulated food containing peanuts for the control of malnutrition, some experts still consider that it may be preferable to have a peanut free alternative because of the frequent contamination of peanut by aflatoxins and because the taste of peanut is not appreciated in many Asian countries[85-88]. Thus, producing and assessing the efficacy of a peanut free RUTF alternative is an important objective.

During an expert meeting organized by WHO, it was agreed that the demonstration of the efficacy of a food for the treatment of acute malnutrition should not be based solely on anthropometric parameters[89]. The effect on other parameters such us body composition, micronutrients status and long term mortality should also be assessed[89]. Thus, in addition to anthropometric endpoints, body composition, anaemia and long term mortality endpoints are important.

Previous to the first randomised study conducted in Zambia, a formal acceptability and tolerance trial was conducted comparing SMS-RUTF and P-RUTF among 45 children aged 4-11 years. The SMS-RUTF was as acceptable as the P-RUTF with no associated adverse effects[90]. For the second randomised trial conducted in Democratic Republic of Congo (DRC), we assumed that the changes made to the formulation would not affect acceptability and tolerability. Thus, no formal acceptability trial was conducted. The results obtained regarding

frequency of diarrhoea, flatulence, vomiting, skin eruption and RUTF intake confirmed that SMS-RUTF was as accepted as P-RUTF (paper in preparation). The only difference observed was in intake and this difference was not of clinical relevance [214g/day (167kcal/kg/day) for P-RUTF versus 199g/day (152kcal/kg/day) for SMS-RUTF in children below 24 months and 276g/day (157kcal/kg/day) for P-RUTF versus 251g/day (139kcal/kg/day) for SMS-RUTF for children 24 months and more). For the present study, it is anticipated that the addition of the AA mixture will modify significantly the taste of the product. It is also well-known that the addition of milk powder makes the product sweeter.

Data on the efficacy of milk free RUTF in treating SAM in children are scarce. Valid has successfully conducted two randomised studies on the subject. These results showed that FSMS-RUTF was effective in children two years and above but not among children less than two years. [91]. Based on the results of that study, the formulation of FSMS-RUTF has been optimised

2.3 Objectives and hypotheses

The goal of this research programme is to assess in two different phases; the acceptability trial (phase 1) and the efficacy trial (phase 2) of two innovative Soya-Maize-Sorghum RUTFs for the treatment of SAM in children under five years old. The two products are the new version of the Milk Free Soya-Maize RUTF (FSMS-RUTF) and the 10% Milk Soya-Maize-Sorghum RUTF (MSMS-RUTF). The new SMS-RUTF has an improved AA profile compared to the previous milk free SMS-RUTF and has less phytic acid. The innovation here is the use of a mixture of AA to improve the AA profile of the product. The AA mixture is added to both study foods: the SMS-RUTF (FSMS-RUTF and MSMS-RUFT).

2.3.1 Specific objectives

Results of the acceptability phase will be used to refine the recipe in term of tailoring the products to preferences of Malawian children and to address factors that have resulted in any side effect observed. This optimisation, if needed, will have to be done prior to the efficacy trial. Efficacy of the new version SMS-RUTFs will be determined based mainly on the recovery rate. The other specific objectives are necessary for describing the quality of nutritional recovery judged by anthropometric criteria.

2.3.1.1 Specific objectives for acceptability trial

For the acceptability trial there are two principal specific objectives and one secondary specific objective.

Primary specific objectives:

1 Compare organoleptic characteristics (taste, smell, texture, colour) of FSMS-RUTF and MSMS-RUTF to that of P-RUTF in 3 to 5 years Malawian children

2 Compare frequency of RUTF related side effects among children taking FSMS-RUTF or MSMS-RUTF to the frequency in children taking P-RUTF.

Secondary specific objective:

3 Compare levels of breath hydrogen excretion among children taking FMSMS-RUTF or MSMS-RUTF to that of those taking P-RUTF.

2.3.1.2 Specific objectives for efficacy trial

The specific objectives (SOs) for the efficacy trial include the two primary objectives (1 and 2) and 16 secondary objectives listed below:

Primary specific objectives:

1. Compare the recovery rate of children aged 6-23 months with SAM treated with FSMS-RUTF with that of those treated with MSMS-RUTF and to that of children of same age treated with P-RUTF
2. Compare the recovery rate of children aged 24-59 months with SAM treated with FSMS-RUTF with that of those treated with MSMS-RUTF and to that of children of same age treated with P-RUTF

Principal secondary specific objectives:

1 Compare weight gain of children aged 6-59 months with SAM treated with FSMS-RUTF with that of those treated with MSMS-RUTF and to that of children of same age treated with P-RUTF (age group 6 to 23 months and 24 to 59 months to be sampled and analysed separately)

2 Compare time to recovery of children aged 6-59 months with SAM treated with FSMS-RUTF with that of those treated with MSMS-RUTF and to that of children of same age treated with P-RUTF (age group 6 to 23 months and 24 to 59 months to be sampled and analysed separately)

3 Compare height gain at 3 months after discharge of SAM children 6 to 59 months treated with FSMS-RUTF or MSMS-RUTF between them and to that of children of same age treated with P-RUTF (age group 6 to 23 months and 24 to 59 months to be sampled and analysed separately)

4 Compare deuterium dilution technique estimated body composition (Body Fat Mass. percentage body Fat Free Mass and lean mass) change at full recovery of children treated with FSMS-RUTF, MSMS-RUTF and P-RUTF

5 Compare change in phase angle over time of children treated with FSMS-RUTF, MSMS-RUTF, P-RUTF.

Other secondary specific objectives:

6 Compare the efficacy of FSMS-RUTF , MSMS-RUTF and P-RUTF in reducing the prevalence of anaemia among children recovering from SAM

7 Compare daily intake of RUTF in grams and in kcal/kg/day of children 6 to 59 months treated for SAM with FSMS-RUTF, MSMS-RUTF and P-RUTF (age group 6 to 23 months and 24 to 59 months to be sampled and analysed separately)

8 Compare breast milk intake of children 6 to 23 months old treated for SAM with FSMS-RUT, MSMS-RUTF and P-RUTF.

9 Compare proportion of children without intestinal inflammation as measured by stools calprotectin at 14 days, 28 days and recovery between SAM children treated with FSMS-RUTF, MSMS-RUTF and P-RUTF.

10 Compare recovery in level of plasmatic level of key growth related amino acid of children treated with FSMS-RUTF, MSMS-RUTF, P-RUTF and community controls.

11 Compare after discharge survival in SAM children treated with FSMS-RUTF, MSMS-RUTF, P-RUTF and community controls.

2.3.2 Hypotheses

The hypotheses for the objectives described above are as follows:

2.3.2.1 Phase 1: acceptability trial

Primary hypotheses:

1 FSMS-RUTF and MSMS-RUTF have organoleptic characteristics (taste, smell, texture, colour) as acceptable as the standard milk and peanut based RUTF (P-RUTF) for Malawian children aged 3 to 5 years attending community based child care centres (CBCC)

2 FSMS-RUTF or MSMS-RUTF intake will not be associated with more RUTF related side effects than P-RUTF in Malawian children aged 3 to 5 years attending CBCC.

Secondary hypothesis:

3 FMSMS-RUTF and MSMS-RUTF will not be associated with a higher level of breath hydrogen excretion when compared to P-RUTF.

2.3.2.2 Phase 2: efficacy trial

Primary hypotheses:

1 Recovery rate of children aged 6 to 23 months with SAM treated with FSMS-RUTF is > 75% and is not inferior to that of children with SAM of the same age treated with MSMS-RUTF nor to that of P-RUTF with the fixed non-inferiority margin being 10%.

2 Recovery rate of children aged 24 to 59 months with SAM treated with FSMS-RUTF is > 75% and is not inferior to that of children with SAM of the same age treated with MSMS-RUTF nor to that of P-RUTF with the fixed non-inferiority margin being 10%.

Principal secondary hypotheses:

1 Weight gain (g/kg/day) during of children aged 6 to 59 months with SAM treated with FSMS-RUTF is not inferior to that of children with SAM of same age treated with MSMS-RUTF nor to that of those treated with P-RUTF with the fixed non-inferiority margin being 1.5g/kg/day (age group 6 to 23 months and 24 to 59 months to be sampled and analysed separately)

2 Time to recovery under treatment from admission to recovery of children aged 6 to 59 months with SAM treated with FSMS-RUTF is within the non-inferiority margin of 14 days when compared to children of the same age treated with MSMS-RUTF or to P-RUTF (age group 6 to 23 months and 24 to 59 months to be sampled and analysed separately).

3 Height gain during recovery and 3 months after discharge of children aged 6 to 59 months (admission age) with SAM treated with FSMS-RUTF is not inferior to that of children with SAM of the same age treated with MSMS-RUTF nor to that of children treated with P-RUTF given a fixed non-inferiority margin of 0.5 cm (age group 6 to 23 months and 24 to 59 months to be sampled and analysed separately)

4 Proportionally to body weight, percentage of body fat mass (BFM) measured at the time of recovery of children treated with FSMS-RUTF will not be higher than the percentage of BFM in children treated with MSMS-RUTF nor higher than the percentage of BFM of those treated with P-RUTF at the fixed margin of inferiority of 6%.

5 Tissues electrical properties assessed by Bio-electrical impedance analysis (BIA) will not be impaired in children treated with FSMS-RUTF when compared to that of children treated with MSMS-RUTF or with P-RUTF given a margin of equivalence to be determined by the synthesis methods based on findings of the SMS-RUTF study recently completed in DRC.

Other secondary hypotheses:

6 Haemoglobin gain of children treated for SAM with FSMS-RUTF will not be lower than that of children treated with MSMS-RUTF nor than that of those treated with P-RUTF given a margin of non-inferiority of 0.5g/dl.

7 The RUTF daily intake in grams and in kcal/kg/day of children 6 to 59 months old treated for SAM with FSMS-RUTF will not be inferior to the daily intake of children of the same age treated with MSMS-RUTF nor to the daily intake of those treated with P-RUTF given a margin of non-inferiority of 18g/day and 25kcal/kg/day, respectively (age group 6 to 23 months and 24 to 59 months to be sampled and analysed separately).

8 Breast milk intake of children 6 to 23 months old treated for SAM with FSMS-RUTF will not be lower than the breast milk intake of children of the same age treated with MSMS-RUTF nor to the intake of those treated with P-RUTF given a margin of non-inferiority of 100g/day.

9 Proportion of children with normalised stool calprotectin at 14 days, 28 days and recovery will not be lower in children treated for SAM with FSMS-RUTF than in those treated with MSMS-RUTF or in those treated with P-RUTF given a margin of non-inferiority determined using the synthesis method based on the confidence interval observed in children treated with P-RUTF.

10 Plasmatic level of key growth related AA of children treated with FSMS-RUTF will not be inferior to that of children treated with MSMS-RUTF or P-RUTF given a margin of non-inferiority defined using the synthesis method

11 12-months after discharge survival in children with SAM treated with FSMS-RUTF will not be inferior to that of children treated with MSMS-RUTF nor to that of those treated with P-RUTF given a margin of non-inferiority of 10%.

2.3.3 Margin of non-inferiority

The margin of non-inferiority was fixed using the margin fixed method except for the secondary specific objectives 8, 15 and 16 of the efficacy trial, for which the synthesis method will be used. The margin for the recovery rate of 10% and of the time to recovery of 14 days were used in our previous study and was accepted by the reviewers of journals that have published the findings [15, 92]. All the other non-inferiority margin stated are based on the 95% confidence interval of the difference observed in our recent SMS-RUTF efficacy trial conducted in DRC (paper in preparation).

2.4 Setting

The research programme will be conducted in the Central Region of Malawi. For the acceptability trial, two CBCCs will be used for the study. We have pre-agreement with several CBCCs but the final choice of the CBCCs will be made in January 2015 based on the functionality and on the number of children attending the centres at that time. The efficacy trial will be carried out in 3 Health District of the Central Region as recommended by the Ministry of Health. These Health Districts are Lilongwe, Dedza and Mchinji. Pre-agreement have been secure with the District Health Officers (DHO) of these Health Districts. In each Health District, a Cluster (a sub-administrative area in Malawi administrative organisation) with the highest burden of acute malnutrition according to the 2014 monthly routine statistics will be selected. In total, 30 community based feeding centres will be established (10 in each District) in the catchment areas of OTP sites that reported more than 60 admissions of SAM cases in 2013. The list will be finalised based on the 2014 statistics and after a field visit has cross-checked the routine statistic figures. The Central region has the worst indicators for undernutrition among under-five children. During the 2010 Malawi Demographic Survey, the prevalence of stunting was 47.2% while that of underweight was 13.5%. The prevalence of wasting is low at 1.8% for severe wasting and 4.3% for wasting. However, this figure underestimates the actual incidence of SAM because up to 80% of cases are oedematous in Malawi and they are not included in these statistics[93]. Indeed, according to

Ministry of Health, by the end of August 25,313 children with SAM had been admitted to CMAM in 2013/2014 consumption year (UNICEF, Malawi situation report, July and August 2014).

2.5 Methods

2.5.1 Study design

2.5.1.1 Acceptability trial

This will be a cross-over trial. In each site, children will be divided in 2 groups. In 2 separate phases, each group will alternatively receive one of the study food (FSMS-RUTF or MSMS-RUTF) and the control food (P-RUTF). The sequences of the phase will be as indicated in the table 1 below:

Table 1: Cross-over sequences.

SITE	ARM	PHASE 1	PHASE 2
NUMBER 1	INTERVENTION	FMSM-RUTF	P-RUTF
	CONTROL	P-RUTF	FMSM-RUTF
NUMBER 2	INTERVENTION	MSMS-RUTF	P-RUTF
	CONTROL	P-RUTF	MSMS-RUTF

As indicated above, the principal endpoints of the acceptability trial are:

1. Acceptability defined by the criteria listed below
    a. Persistence of good appetite
    b. Consumption of more than 75% of the offered food within 1 hour. (meal acceptance)
    c. Consumption of more than 75% of the offered food for more than 75% of the days on trial (overall acceptance)
    d. Consumption of a quantity of energy not less than 75% of the energy intake from the control food.
2. Tolerance that is confirmed if:
    a. ill-effects associated with the food requiring withdrawal from the trial is less than 10% and if the
    b. The difference in the frequency of side-effect events between the intervention group and the control group is less than 10%

The prevalence of carbohydrate malabsorption that will be determined by hydrogen breath test will not be used to define acceptability.

2.5.1.2 Efficacy trial

As stated in the hypotheses the main study will be a 3 group statistician blinded, randomized and controlled, parallel groups, **efficacy** non-inferiority trial. Because the participants will be able to distinguish the different RUTFs based on colour and mouth feel, participants will not be blinded. We will use the day care approach to ensure that children eat only the RUTF of the group to which they have been assigned. No study food will be taken home and the intake will be supervised by study enumerators who will in person feed the children.

The principal endpoints for the efficacy trial are:

1. Recovery rate
2. Mean weight gain
3. Mean time to recovery
4. Mean height gain
5. Percentage of body fat mass at recovery

6. Mean phase angle
7. Prevalence of anaemia
8. Incidence of deaths after discharge

The definition of principal endpoints is given below

(a) Recovery rate

The recovery rate will be measured as a percentage of those children who are discharged cured from the OTP programme divided by the total number of children who exit from the programme

$$\text{Recovery rate} = \frac{\text{No. children discharged Cured}}{\text{Total No. of Exit}} \text{ X } 100$$

(b) Average weight gain

The average weight gain will be measured as the mean of the growth velocity expressed in g/kg/day of individual children calculated as follow:

$$\text{Weight gain} = \frac{\text{Discharge weight (g)} - \text{Admission weight(g)}}{Admission\ weight(kg) * Length\ of\ stay(days)}$$

$$\text{Average weight gain} = \frac{\Sigma \text{ weight gains}}{Total\ children}$$

(c) Average time to recovery or Length of stay

The average length of stay (LOS) is the mean of the LOS expressed in days of individual children calculated as follow:

$$\text{Average LOS} = \frac{\Sigma(\text{Discharge date} - \text{Admission date})}{Total\ of\ children}$$

(d) Average height gain

The average height gain is the mean of the height gain expressed in centimetres of individual children calculated as follow:

$$\text{Average height gain} = \frac{\Sigma(\text{Height 3 months after discharge (cm)} - \text{Admission height (cm)})}{Total\ of\ children}$$

(e) Prevalence of anaemia

The prevalence of anaemia will be expressed in percentage and calculated as follow

$$\%\ anaemia = \frac{\text{n haemoglobin} < \frac{11.0\text{g}}{\text{dl}} * 100}{\text{total children with results}}$$

(f) Incidence of death after discharge

This endpoint will be expressed in person-months and calculated as follow:

$$incidence\ of\ deaths = \frac{\text{n deaths}}{\sum \text{time of observation}}$$

(g) Percentage body fat mass

Percentage of body fat will be calculated by the laboratory performing the analysis. After determining total body water and total lean mass, the total body fat mass will be obtained by subtracting the lean mass to the total body weight. Then percentage body fat will be calculated as follow:

$$\%Body\ Fat\ mass = \frac{\text{Calculated Body Fat Mass(kg)} * 100}{\text{Body weight(kg)}}$$

(h) Phase angle

The phase angle will be provided by the BIA device.

2.5.2 Study populations

2.5.2.1 Acceptability trial

Children aged 3 to 5 years regularly attending the selected CBCC with signs of moderate acute malnutrition as defined by MUAC between 11.5 and 12.5 cm will be eligible for the acceptability trial. The other eligibility criteria include absence of bilateral pitting oedema, absence of any disability, absence of any acute illness, absence of skin lesions, absence of mouth lesions, permanent resident of the area residing at a maximum radian of 30 minutes walk with no plan to travel during the period of the study, and parent or caregiver consent. Two CBCC will be used for the study. Each site will be testing a separate study food.

2.5.2.2 Efficacy trial

The eligibility criteria for inclusion into the study are:

- Age between 6 months and 59 months
- Presence of SAM defined by MUAC<115mm or presence of bilateral pitting oedema of any grade or presence of clinical signs of wasting confirmed by the supervisor.
- Residing within 1 hour walking distance from the day-care centre
- Caregivers consent to participate

The exclusion criteria for children meeting these criteria include:

- Presence of disability including congenital or acquired disorders affecting growth
- Refusal to participate (children who refuse to participate will be referred to the existing outpatient therapeutic feeding sites not participating into the study)
- Children of visiting families who risk to return to their location before completing the study that include the therapeutic feeding phase and follow up after discharge phase. These children will also be referred to the non participating outpatient therapeutic feeding sites.
- Children with history of any food allergy
- Child with previous admission for severe acute malnutrition in the previous 3 months (will be referred to non participating outpatient therapeutic centres)

Children who present with SAM with complications will be first referred for inpatient care and enrolled into the study after stabilisation if they meet eligibility criteria. Children not assessed by study team prior to admission into the stabilisation centre will not be eligible. Children with HIV or tuberculosis meeting criteria for outpatient management will be linked to the nearest health facility providing comprehensive treatment of these diseases and included into the research despite the presence of these chronic infections. The research team will ensure adherence to both the medical treatment and the nutrition treatment.

2.5.2.3 Community controls

Community controls will be children with height/length-for–age Z-score ≥-1, no wasting and, no chronic disease or disability who are stable resident of the study area and who has no plan to move from the area. They will be recruited from the neighbourhood of children they will be matched with or among children attending the same CBCC. Controls for the body composition sub-study and for the breast-milk intake sub-study, will be

recruited consecutively to the admission of a SAM case into the sub-study. For the post-discharge survival the matching will be done prospectively as children are discharged from the therapeutic feeding phase. The matching ratio will be 1 case for 1 community control for both objectives. The matching criteria will be age (± 3 months) and gender.

2.5.3 Sample size and randomisation

2.5.3.1 Sample size

For the acceptability trial, a convenience sample of 20 children per group will be used. For assessing efficacy, the different sample sizes are calculated for a power of 80% and a level of significance of 0.05. The total sample size will be the total of sample required for the SO1 and SO2. For the other SOs, the calculated sample was lower than that required for the total sample for SO1 and SO2 as indicated in table 2 below.

Table 2: Sample size according to the specific objective for the efficacy study

Specific objective	endpoint	Research design	Assumptions	Calculated sample
SO1	Recovery rate<br>6-23 months	Non-inferiority clinical trial	Recovery rate<br>P-RUTF, FSMS-RUTF, MSMS-RUTF= 75.5%<br>Non-inferiority margin=10%<br>Loss to follow up =10%	250 per arm.<br>Total=750 SAM children
SO2	Recovery rate<br>24-59 months	Non-inferiority clinical trial	Recovery rate P-RUTF, FSMS-RUTF, MSMS-RUTF = 88.2%<br>Non-inferiority margin=10%<br>Loss to follow up =10%	150 per arm.<br>Total=450 SAM children
SO3	Mean<br>weight gain<br>6-23 months	Non-inferiority clinical trial	Standard deviation=4.1<br>non-inferiority margin =1.5g/kg/d<br>Loss to follow up=10%	110 children/arm<br>Total=330 SAM children
SO4	Mean<br>weight gain<br>24-59 months	Non-inferiority clinical trial	Standard deviation=3.5<br>non-inferiority margin =1.5g/kg/d<br>Loss to follow-up=5%	75 children/arm<br>Total=225 children
SO5	Mean<br>Length of stay<br>6-23 months	Non-inferiority clinical trial	Standard deviation=22.8<br>non-inferiority margin = 14 days<br>Loss to follow up=10%	40 children/arm<br>Total= 120 children
SO6	Mean<br>Length of stay<br>24-59 months	Non-inferiority clinical trial	Standard deviation=9.9<br>non-inferiority margin = 14 days<br>Loss to follow up=10%	30 children/arm<br>Total= 90 children
SO7	Mean<br>Fat free mass (%)	Non-inferiority clinical trial	Standard deviation=1.62<br>Margin of non-inferiority = 1.07kg<br>Loss to follow up=5%	35 per arm<br>Total=105 children<br>6-59 months
SO8	Mean<br>Phase Angle	Non-inferiority clinical trial	Standard deviation=0.7<br>Margin of non-inferiority = 0.2<br>Loss to follow up=10%	170/arm<br>Total= 510 children<br>6-59 months
SO9	Mean<br>height gain<br>3 months after discharge	Non-inferiority clinical trial	Standard deviation=0.7<br>Margin of non-inferiority = 0.5cm<br>Loss to follow up=10%	180 /arm<br>Total=540 children<br>6-23 months
SO10	Mean<br>height gain<br>3 months after discharge	Non-inferiority clinical trial	Standard deviation=0.8<br>Margin of non-inferiority = 0.3 cm<br>Loss to follow up=10%	100 /arm<br>Total=300 children<br>24-59 months
SO11	Anaemia rate	Non-inferiority	Standard deviation=1.3<br>Non-inferiority margin = 0.5 g/dl<br>Loss to follow up=10%	100 /arm Total=300 children (both age group)

The sample size for non-inferiority assessment is calculated using the software “Power” available at http://www.sealedenvelope.com/power binary noninferior.php for binary endpoint and http://www.sealedenvelope.com/power_continuous_noninferior.php for continuous endpoint. For binary endpoint, the software uses the formula $n = f(\alpha, \beta) \times [\pi s \times (100 - \pi s) + \pi e \times (100 - \pi e)] / (\pi s - \pi e - d)^2$ where πs and πe are the true percent 'success' in the standard and new (experimental) treatment group respectively $f(\alpha, \beta) = [\Phi\text{-}1(\alpha) + \Phi\text{-}1(\beta)]2$ and Φ-1 is the cumulative distribution function of a standardised normal deviate [94]. For the continuous endpoint, the Calculation is based on the formula: $n = f(\alpha, \beta) \times 2 \times \sigma 2 / d2$ where where σ is the standard deviation and $f(\alpha, \beta) = [\Phi\text{-}1(\alpha) + \Phi\text{-}1(\beta)]^2$ [95].

2.5.3.2 Randomisation

The study will use simple randomisation and the sealed opaque envelope method will be used for the final allocation of group.

For the acceptability trial, using a pre-established list of sites, eligible children with their caregivers will be invited one by one and asked to pick an envelope containing the study group from a sealed box. No change of envelope will be allowed.

For the efficacy trial, to minimize selection bias and maximize chances of having balanced allocation in all the 3 study groups, a block randomisation with random block size scheme will be used. Thus, in blocks of different sizes (the size will be decided in advance and randomly), children will be prospectively allocated to one of the 2 intervention groups (FSMS-RUTF group or MSMS-RUTF group) or the control group in which the standard RUTF (P-RUTF) will be used. Different randomisation list will be established for 6 to 23 months and 24 to 59 months children.

2.5.4 Study implementation approach and procedures

2.5.4.1 Both components: anthropometric procedures

2.5.4.1.1 Weight

Weight will be measured at the same time of day at the arrival to the day-care centre. For each site, the weighing will be done by the same team of two persons throughout the study, monitored and supervised closely by the same field research supervisors. All children will be weighed undressed. The weighing will be done following WHO recommended procedures (Training Course on Child Growth Assessment. Geneva, WHO, 2008). For children less than 2 years old or unable to stand, we will do tared weighing. For children 2 years or older, we will weigh the child alone if the child can stand still.

2.5.4.1.2 Height/Length

The height/length will be done following WHO recommended procedures (Training Course on Child Growth Assessment. Geneva, WHO, 2008). A locally made wood height/length board will be used for the measurement. Depending on a child’s age and ability to stand, we will measure the child’s length (length is measured lying down) or height (height is measured standing upright). Children will be measured with height board placed on a flat, stable surface such as a table for length or against a straight, vertical surface such as a wall or pillar for height with the head, back, buttocks, and heels touching the backboard; heels together; knees extended; and head in the Frankfort horizontal plane. The measurement will always be done by two researcher officers. One person will be considered as principal measurer and the second as his assistant.

2.5.4.1.3 MUAC

MUAC will be measured using a standard MUAC tape. The measurer will locate the tip of the child’s shoulder and bent the elbow to the right angle, mark the midpoint of the arm by measuring from the tip of the shoulder

and tip of the elbow, straighten the child's arm and wrap the tape around the arm at the mid-point, make sure the numbers on the tape are right side up and tension is not too tight and too loose.

2.5.4.2 *Acceptability trial*

2.5.4.2.1 Intake monitoring

The Intake during both phases will be monitored by the research team. Children will attend the feeding point according to the usual opening hours of CBCCs. During the stay at the feeding point of the CBCC, children will be offering a 92g sachet of RUTF that they should consume in 3 servings. They will be offered the possibility to ask for more if they finish their sachet. Each meal intake will be recorded and the specific information collected will include:

- quantity given
- quantity eaten
- quantity of waste or spillover
- eating pattern (food refusal, need for support and insistence to eat, duration of the meal)
- request of additional amount

2.5.4.2.2 Anthropometry and morbidity data

At the time of admission, baseline data on weight, mid upper arm circumference, appetite, illnesses symptoms and signs, rash and growth pattern will be collected. Subsequently, weight will be measured daily before the first meal while the mid upper arm circumference will be measured weekly. The height will be measured once at admission. A 24-h recalls of the presence of the following symptoms will be carried out daily:

- Fever
- Change of mood
- number of stools/ 24 hours (History from the caretakers and observation during presence at the feeding point)
- vomiting : number of time for the previous 24/hours (8:00 to 8:00)
- Complain of abdominal pain
- Abdominal distension
- Rashes
- Cough
- All other observed symptoms will also be recorded.

A qualitative assessment of other food consumed by the child during the 24 hours will also be recorded daily.

2.5.4.2.3 Liking and disliking rating

During each phase, the hedonic scale will be used twice to assess child feeling regarding the RUTF. The first session of assessing the liking or disliking will be done at the beginning of the phase and the second will be done after 7 days of intake. Three methods will be used for ascertaining the rating: facial observation by a trained research officer, showing pictures of the different rating to the child for him to select the appropriate picture and when appropriate by asking the child to verbalise his feeling. Each child will be asked daily to confirm his willingness to continue taking the RUTF.

2.5.4.2.4 Hydrogen breath test

The hydrogen breath tests will be done using the Gastrolyzer+® to measure carbohydrate malabsorption. The test will be realised the last day of the phase for a sub-sample of children enrolled in the acceptability trial. The day before the test, mother will be advised to not give breakfast to the child and arrive as early as possible on the site. A baseline breath measurement will be taken upon arrival at the CBCC following which each child will receive a sachet of the food of his study arm and phase. Subsequently the hydrogen concentration in the exhaled air will be measured every 15 minutes for first hour and then every 30 minutes for the next three hours. At each interval, the hydrogen concentration will be measured in triplicate.

Carbohydrate malabsorption will be diagnosed if either or both of the following conditions are met: 1) ≥10 ppm increase in hydrogen production above baseline after one hour or 2) hydrogen production of >20 ppm after one hour [19].

2.5.4.3 Efficacy trial

2.5.4.3.1 Treatment approach

RUTF is mostly used for the outpatient management of SAM and children are normally fed with RUTF by their main caregiver at home. However, to ensure that the research team has full control over the daily RUTF intake of study children**, a day care approach** will be used. The use of the day-care approach will eliminate the potential effect of operational constraints such us sharing, or sale of RUTF and inappropriate intake such us mixing RUTF with other foods that dilute energy and micronutrient densities. Although the use of the day-care approach may increase absenteeism, it will help to categorize children into those who strictly adhered to the protocol and those who did not adhere. It will also help to quantify the average daily intake from RUTF at individual level during the whole period of nutrition rehabilitation. An attempt will be made to capture the intake of other foods that children may eat at home in the evening.

2.5.4.3.2 Treatment protocol

Nutrition and medical management of children of both study groups will be similar and will follow the national guidelines for the management of moderate and severe acute malnutrition in children with a slight change in discharge criteria as described in the table 3 below. The only difference of management between the 2 groups will be the type of therapeutic food. Children will be prescribed 200 kcal/kg/day of either SMS-RUTF (intervention group) or P-RUTF (control group). They will be fed this amount in 4 servings at the day care centre between 9 am and 3 pm.

Table 3. Admission and discharge criteria for 6 to 59 months old children

**Admission**	**MUAC < 115mm**<br>**Bilateral pitting oedema grade + or ++**<br>**WFH Z-score <- 3 (WHO 2006 curves)**
**Discharge**	**Absence of bilateral pitting oedema for 14 successive days**
	**MUAC>=125mm for 14 successive days**
	**Absence of acute life threatening infection**

2.5.4.3.3 Bioelectrical Impedance Analysis

The bioelectrical impedance analysis (BIA) measurements using the Bodystat 1500 MDD system (Bodystat Inc, Douglas, United Kingdom). Children will be measured while lying in supine position with arms and legs slightly abducted from the trunk. The measurement will start after 3 to 4 minutes in that position and will be done with electrodes placed at the dorsal surfaces on wrist (between second and third metacarpals) and ankle (between second and third metatarsals) as placing them only 1cm off the imaginary line of wrist and ankle will affect the percentage of FFM and FM substantially. The proximal and distal electrodes will be a minimum of 5 cm apart. The impedance will be measured at two frequencies (50 KHz and 5 KHz) and the ratio of measured impedance at 50 KHz and 5 KHz also called wellness marker (WM) will be calculated. Phase angle (PA) is related to the resistance (opposition offered by the body to the alternating current) and to the reactance (the variation in the phase of the alternating current due to the conductivity capacity of the cells membranes) and expresses both changes in the amount as well as the quality of soft tissue mass (cell membrane permeability and

soft tissue hydration). Thus, PA is an indicator of membrane integrity and water distribution between the intra- and extra-cellular spaces. For this study the PA calculated by the Bodystat 1500 MDD will be used. Well-nourished age and gender matched from the community will serve as control.

2.5.4.3.4 Body composition using the Deuterium dilution technique

Body fat will also be estimated by the deuterium dilution technique (DDT) at admission and 3 month after recovery. The exam will be done in a child well hydrated with empty bladder. A dose of deuterium of 3g (children <10kg) or 6g (children 10 to 20 kg) will be given in the morning after an overnight fasting, Saliva samples will be collected before giving the deuterium dose (baseline sample) and 3 hours (post-dose 1) and 4 hours (post-dose 2) after taking the dose. The saliva sample will be collected using the technique of cotton wool ball. Participants will avoid eating, drinking and physical activity until the final saliva samples have been taken. After collection, the samples will be stored in a cool box until transferred to a freezer where they will be stored frozen (at –20ºC) until analysis. The enrichment of deuterium in saliva samples will be measured by Fourier Transform Infared (FTIR) spectrometry at the Kenya Medical Research Institute (KEMRI). Well-nourished age and gender matched from the community will serve as control.

2.5.4.3.5 Determination of breast-milk intake

Breast milk intake will be determined by a deuterium oxide-dose-to-the-mother method as described previously[96]. Mothers of participating children will be asked to drink a dose of 30g of deuterium oxide (99.8% purity) after the collection of baseline saliva for the mother and the child. Mother/child post-dose saliva will be collected on days 1, 2, 3, 4, 13 and 14. Saliva samples will be analyzed for deuterium oxide enrichment using Fourier Transformed Infrared Spectrophotometer at the Kenya Medical Research Institute (KEMRI). Well-nourished age and gender matched from the community will serve as control.

2.5.4.3.6 Determination of haemoglobin concentration

The Haemoglobin concentration will be determined at baseline and at discharge using capillary blood collected by finger prick with portable HemoCue®AB spectrophotometer (Ängelholm, Sweden).The device will be calibrated on daily basis using a HemoCue Control Cuvette.

2.5.4.3.7 Determination of amino acid profile

Plasma samples for amino acid analysis will be obtained using EDTA as an anticoagulant. Blood samples will be immediately stored on wet ice to prevent micro-hemolysis and transported within 4 hours to the University of North Carolina (UNC) project laboratory for centrifugation and stored maximum at −20°C until shipping in bulk to the laboratory abroad. Measurement of amino acid concentrations will be performed using an automatic amino acid analyser at Ajinomoto Lab in Japan.

2.5.4.3.8 Determination of stools calprotectin

A single child stools sample collected in a plastic pot upon arrival at the CBCC site at admission, 28 days after starting treatment and at discharge will be used. Faecal calprotectin will be determined using a quantitative point-of–care chromatographic immunoassay kit (Quantum Blue®, Alpha laboratories, Hampshire, UK). As per manufacturer's instructions, two drops of homogenised stool will be applied inside a plastic cassette and the cassette inserted into a portable electronic reader that will display numerical value representing calprotectin concentration. The cassette reader will be calibrated for each day of tests.

2.5.4.3.9 Determination of other biological parameters

At admission and discharge, venous blood sample will also be collected for measurement of acute inflammation (CRP, ESR, and inflammatory cytokines), biological protein indicators (albumin), lipid profile (cholesterol and triglycerides), liver function (ALT, AST) as well as micronutrient status (iron, ferritin and zinc) of the enrolled children. Blood samples will be collected using sterile and disposable butterfly needle by a trained phlebotomist from the paediatric ward of the Baylor Clinic located at Lilongwe[97]. For the analysis to be performed in Malawi, sample management, transportation and preparation for analysis will done according to the standard

procedures as per World health Organisation guidelines and according to procedures used by the Malawi laboratory of the UNC project. For analysis to be performed abroad, samples will prepared for storage and stored according to the requirement of each test. Samples will stored at -70°C until analysis or if not possible at -20°C. Samples post analysis will also be stored to allow for future analyses. The duration of pot-test samples storage will be 5 years.

2.5.4.4 Data to be collected

Data will be collected on a specially designed study form that will be adapted from the standard Outpatient Therapeutic Programme form included in the national guidelines for the management of acute malnutrition. The form will record administrative, nutrition and morbidity data at admission, during recovery and at discharge. The table 4 below provides an overview of the data to be collected and the schedule of their collection.

Table 4: Data to be collected at admission, during nutrition rehabilitation and during follow up after discharge

Variable	Schedule										
	Admission	During nutrition rehabilitation					Follow up after discharge				
		daily	Weekly	Bi-weekly	Discharge	other	M3	M6	M9	M12	other
**Socio-demographic data**											
Adress	X				X		X	X	X	X	
Mother socio-demographic	X				X		X	X	X	X	
Father socio-demographic	X				X		X	X	X	X	
Household data	X						X	X	X	X	
Natural hazard/Disaster						WO					WO
Man made hazard/Disaster						WO					WO
**Nutrition data**											
**Oedema**	X	X					X	X	X	X	
Weight	X	X					X	X	X	X	
MUAC	X	X	X				X	X	X	X	
Height	X			X			X	X	X	X	
RUTF intake/appetite	X	X									
Home food intake	X	X									
Hemoglobin	X				X						
Body fat mass	X				X	W2					
Fat Free mass	X				X	W2					
**Bioelectrical Impedance Analysis**											
Phase Angle	X				X	W2					
Welness Marker	X				X	W2					
**clinical and morbidity data**	X										
Fever	X	X									
Cough/fast or difficulty in breathing	X	X									
Diarrhea/vomiting/dehydration	X	X									
Adbdominal pain	X	X									
Flatulence	X	X									
Skin eruption	X	X									
antibiotic prescription	X	X									
Malaria parasites	X	X		X		Fever					
Stools parasites		X				Diarrhea					
HIV test	X	X									
Tuberculosis diagnosis	X	X									
**Discharge data**											
Type of Discharge				X							
Date				X							
Vital status after discharge (mortality)							X	X	X	X	X
**Community controls**											
MUAC					X						
Weight					X						
**Height**					X						
Body fat mass					X						
Fat Free mass					X						
**Phase Angle**					X						
Wellness Marker					X						
Vital status (mortality)/verbal autopsy					X						

2.5.4.5 Data recording

Data will be collected on a standardised Outpatient Therapeutic Programme forms adapted from the National OTP cards. Individual patient data will be double entered into a database specially designed for this study by two different data entry clerk. Field supervisors will cross-check data collection forms for completeness and filling quality and will check data entry quality by checking 10% of the entry of the month.

2.5.5 Therapeutic foods and patient care

The table 5 below provides the composition of all study RUTFs. All study foods will be produced in the Valid Nutrition factory in Malawi that is officially recognized as a UNICEF approved supplier for RUTF. The Factory is supplying the Ministry of Health of Malawi since 2005 and has produced study foods for several published studies.

Table5: Composition of the study therapeutic food

	FSMS- RUTF	MSMS-RUTF	P-RUTF
Total content (g)	100.0	100.0	100.0
Content witout MN (g)	98.0	98.0	98.4
Cost (US$)*1	0.13	0.17	0.26
Energy (kcal)	520.0	520.0	526.8
Protein (g)	15.6	15.6	14.7
Fat (g)	34.3	34.5	33.1
Carbohydrate(g)	42.1	40.7	46.6
Fiber(g)	5.0	5.0	1.5
Sodium (mg)	5.3	56.3	258.1
Potassium (mg)	1155.8	1155.8	1140.0
Calcium (mg)	437.8	437.8	328.6
Phosphorous (mg)	446.0	446.0	370.0
Magnesium (mg)	166.8	100.1	86.0
Iron (mg)	60.0	60.0	12.0
Zinc (mg)	20.6	20.6	11.1
Copper (mg)	1.4	1.4	1.7
Selenium (ug)	20.0	20.0	17.5
Iodine (ug)	82.3	82.3	100.0
Vit A-RAE (ug)	995.0	995.0	910.0
Vit D (mcg)	15.0	15.0	16.0
Vit E (mg)	40.7	40.7	20.0
Vit K (ug)	41.9	41.9	21.0
Thiamin (mg)	1.5	1.5	0.6
Riboflavin (mg)	2.8	2.8	1.8
Vit C (mg)	329.0	329.0	53.0
Vit B6 (mg)	3.0	3.0	0.6
Vit B12 (ug)	4.3	4.3	1.8
Folate (ug)	200.0	200.0	210.0
Vit B3 (mg)	18.8	18.8	5.3
Vit B5 (mg)	4.5	4.5	3.1
Vit B7 (ug)	60.0	60.0	65.0
n-6 FFA	5.8	3.4	4.8
n-3 FFA	0.5	0.2	0.1
n6 (%/energy)	10.0	5.9	8.1
n3 (%/energy)	0.8	0.3	0.1
Phytic/zinc molor ratio	3.3	2.2	2.2
Phytic/Fe molor ratio	1.0	0.6	1.9

*1: The Cost is not included MNP
The cost was calcuated using price lisf from Valid Nutrition

2.5.6 Statistical analysis

2.5.6.1 Acceptability trial

For the acceptability trial, descriptive analyses will be performed and used for the conclusion on acceptability and tolerance. The conclusion will made based on fulfilment or not of the set acceptability criteria including sufficiency of intake and frequency of side effects. For the conclusion on children preference, comparison of the median score obtained for each organoleptic characteristics will be made.

2.5.6.2 Efficacy trial

2.5.6.2.1 Statistical analysis strategy

Data will be analysed by a statistician blinded as to type of food of the different group allocation. Both the intention-to-treat (ITT) and Per Protocol (PP) analyses will be used for data analyses. However, as per the recommendation of the CONSORT group, in case of opposition between conclusions from the ITT and PP analyses, the final conclusion will be based on PP analyses as ITT analyses inflate type-I error.

Appropriate quantitative methods will be used to provide answers to the study questions. As recommended by Piaggio et al. of the CONSORT (Consolidated Standards of Reporting Trials) group, the assessment of non-inferiority, the confidence interval approach will be used for the interpretation [98]. The figure 1 below illustrates the interpretation.

Figure. Possible Scenarios of Observed Treatment Differences for Adverse Outcomes (Harms) in Noninferiority Trials

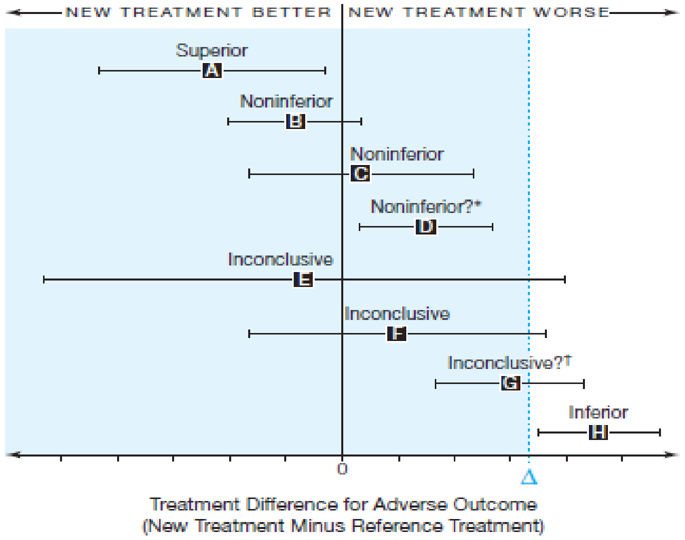

Error bars indicate 2-sided 95% confidence intervals (CIs). Tinted area indicates zone of inferiority. A, If the CI lies wholly to the left of zero, the new treatment is superior. B and C, If the CI lies to the left of Δ and includes zero, the new treatment is noninferior but not shown to be superior. D, If the CI lies wholly to the left of Δ and wholly to the right of zero, the new treatment is noninferior in the sense already defined, but it is also inferior in the sense that a null treatment difference is excluded. This puzzling case is rare, since it requires a very large sample size. It can also result from having too wide a noninferiority margin. E and F, If the CI includes Δ and zero, the difference is nonsignificant but the result regarding noninferiority is inconclusive. G, If the CI includes Δ and is wholly to the right of zero, the difference is statistically significant but the result is inconclusive regarding possible inferiority of magnitude Δ or worse. H, If the CI is wholly above Δ, the new treatment is inferior.[22,43]

*This CI indicates noninferiority in the sense that it does not include Δ, but the new treatment is significantly worse than the standard. Such a result is unlikely because it would require a very large sample size.

†This CI is inconclusive in that it is still plausible that the true treatment difference is less than Δ, but the new treatment is significantly worse than the standard.

From Piaggio et al, JAMA, 2006(295):1152-1160.

For the comparison of survival after discharge from the therapeutic feeding programme, the Kaplan-Meier method will be used to construct survival curves, and the log-rank test and a Cox-regression model will be used

to compare the survival of children who recovered from SAM to that of the community control and assess the effect of some socio-economical and clinical characteristics such us gender, age at discharge, nutrition status at admission and at discharge, HIV status, tuberculosis, and phase angle on survival. Interactions of all the outcomes with the variables gender, form of SAM (oedematous or non-oedematous) and HIV status will be checked.

Given that the study will recruit over a short period of 6 months, no interim analyses will be conducted. However, the independent data monitoring committee will have the right to request Interim analyses if there are reports of several cases of serious side effects.

2.6 Ethical considerations

The study will be conducted in compliance with the Declaration of Helsinki. Permission for the trial to be conducted will be obtained from the Malawi National Health Sciences Research Committee and the Ajinomoto institutional review board. Participation will be voluntary. At the time of admission, each child's parent or carer will be informed about the nature and purpose of the study and asked for their verbal and written consent for their child to be included in the study and for their medical information to be used for research purposes. The caregivers will be informed that participation is voluntary and this information will be emphasised again on the information sheet. The carer information sheet and the consent form will be in English but will also be translated in Chichewa, the national language. The sheet will contain information on the study objectives, study procedures, anticipated duration of participation, potential risks and discomfort for the child, benefits for the child and for the under five population in general, conditions of study termination or participant withdrawal, confidentiality of records and contact persons if any question or problem arise. When parents or carer withhold consent for participation, children will be referred to non participating clinics providing care for SAM children. There are several such clinics in the selected Districts. They use standard P-RUTF. Any child who will develop study food related side effects or any serious disease during the study will be referred to the appropriate level of care and the cost of the treatment met by the research project.

Children participating in the acceptability trial will all receive Vitamin A and deworming drug. Any common illness that occurs during the study will also be treated and any cost not covered by the Government of Malawi scheme will be met by the research programme. Children with SAM admitted to the efficacy trial will benefit from the study interventions. When treated inappropriately SAM is associated with case fatality rates ranging from 20% to 30%. There is good evidence of the benefit of RUTF in reducing case-fatalities among children suffering from SAM, with its use in CMAM programmes reducing SAM-associated mortality from 20-30% to below 5% while increasing programme coverage from below 10% to around 50%. Thus, participating children will have an increased chance of survival and the results may contribute to the overall millennium development objectives of reducing under five mortality. Other benefits for participating children will include free medical care and nutrition and health counselling during the follow up. Children enrolled will receive free appropriate clinical care for all diseases including those not related to the intervention. Children diagnosed with HIV will be linked to the nearest governmental health facility providing specialised care for this disease and the transport costs for travelling to the health facility for specialised follow up will be covered throughout the study period. The benefit for community controls will include Vitamin A supplementation and deworming when needed, free treatment of illness identified during the assessment for inclusion and data collection including anaemia treatment, satisfaction of contributing to identifying solutions for addressing acute malnutrition in Malawi and the world as it has been for the CMAM and a soap as contribution to improving water sanitation and hygiene practices. Transport compensation will be given to the caregivers for each trip to participate to the activity.

2.7 Independent Data Monitoring Committee

A committee of experts will be appointed to serve as an independent data monitoring and safety committee (DMC). The committee will include one academic, a representative of Ministry of Health who are not part of the research team, a member of the National Health Sciences Research Committee and a statistician not part of the research team. The committee will be appointed by the research team and the sponsor with approval from

the Nutrition Unit of the Ministry of Health. DMC will be chaired by the representative of the National Health Sciences Research Committee. The DMC will be notified monthly of the trial's progress and all the cases of study related side effects will be reported to the DMC as they are diagnosed. The DMC will meet at the beginning of the study and twice during the course of the study. However, the chairman of the committee will have the power to call for an extraordinary meeting in case of any report that is considered alarming including occurrence of a serious event due to study food. Indeed, any side effects judged serious will be immediately reported by phone and email to the DHO of the district, the chairman of the DMC and the principal investigators. The principal investigator will report on DMC meetings and decision to Ajinomoto and Valid Nutrition. The chairman of the DMC will have the right to decide an early discontinuation based on the committee risk/benefit assessment.

2.8 Risks and discomforts

Similar products have been used in Zambia and Democratic Republic of Congo in children of same age with the same health condition. There was no particular serious side effect observed. Thus, in the present study there will be minimal risk to the children. The following are the potential risks and the steps that will be taken to minimize these risks:

- The risk of raw and processed food material contamination with bacteria that can cause diarrhoeal illness. Samples will be analysed to ensure that there is no contamination before the food is dispatched at study sites.
- Food allergies and food sensitivities: allergic reaction to the consumption of peanut has been reported in some countries but not in Malawi. If the children have history of food allergy, they will not be asked to participate.
- Suppression of appetite has been reported for some types of legume and cereal based RUTFs. As per our experience in Zambia and DRC, no such effect occurred with the use of SMS-RUTF and therefore we are not expecting to see this effect in Malawi. However, if this does happen to the children, they will be immediately withdrawn from the study and referred to the health facility for the standard treatment.
- Some children can have loose stools at the beginning of the nutrition therapy with RUTF, especially RUTF containing milk. Usually, the situation resolve spontaneously after one or two days and it does not result to any dehydration and does not delay recovery. In case the children do present such symptoms, they will be monitored carefully and given the appropriate additional treatment if required.
- Study foods have a higher level of iron than the standard food. There is a small risk of some abdominal discomfort at the start of the intake due to the level of iron in the food. However, such complaint was not registered in Democratic Republic of Congo where the RUTFs had the same level of iron. Thus, the risk of this effect occurring is low. If the effect does occur the children will be monitored carefully and given the appropriate additional or alternative treatment if required.
- Blood (3 to 5 ml) will be collected by trained nurses wearing sterile and disposable gloves. Each child sample will be taken with a sterile butterfly needle of appropriate gauge for child age and in vacutainer tubes. Gloves and needles will immediately be disposed of appropriately. Taking the sample will produce a small amount of pain equivalent to a pin prick or a prick similar to the one felt when a sample for malaria test is being collected. The pain experienced will be minimised by thorough training of the tester. All blood samples would be handled in a manner that meets good clinical practices standards[97].

2.9 Emergency procedures and contacts

The District Medical Officers will serve as emergency contacts for their respective districts. All the study staffs and the participants will have on the staff or participant information sheet, guidance on how and means of reaching the DHOs in case of study related emergency including their names (Dr Mwayi Mwale for Lilongwe, Dr Salomon Jere for Dedza and Dr Chimwemwe Banda for Mchinji), private and office phone numbers (0888523773 for Dr Mwayi Mwale; 0881188734 for Dr Salomon Jere; 0888528642 for Dr Chimwemwe Banda) and the physical address of the office. After being contacted, DHO will give you advice over the phone and will arrange that a qualified member of the study team visits the child within the next 24 hours and ensure that the child received the best possible care for his condition.

2.10 Confidentiality and dissemination of findings

Confidentiality of each study participant will be maintained at all times through the allocation of a unique identification number that will be used in the electronic database storing the data. Sufficient attention will be paid to protect the rights and individual information of subjects.

A final report will be produced at the end of the research. Other means of dissemination will include presentation of findings to relevant policy makers, presentation in local and international meetings and publication in peer reviewed scientific journals. The journals that will be targeted include The American Journal of Clinical Nutrition, The Lancet, Maternal and Child Nutrition and PLOS Medicine. During the process of publication sufficient attention will be paid to protect the rights and individual information of subjects.

2.11 Data ownership and access

The Government of Malawi own the data and will retain the right to oppose any utilisation of the data that it considers inappropriate, not covered by the ethical approval or that the country authorities regard as misrepresenting the facts. Valid Nutrition and Ajinomoto the sponsor, will have free access to the data, including data of samples analysed abroad. They will have equal right in the management and utilisation of the data.

2.12 Schedule

For the acceptability trial, each child will be followed up for 6 consecutive weeks that include a period of washout of two weeks during which no measurement is taken. For the efficacy trial, children will be followed from the start of treatment until cure that takes approximately 2 months but will be 3 months for the non-respondents. Children eligible for follow up after discharge will be visited twice after the treatment ended. This will be 3 months and 12 months after the end of treatment. Consequently, the study will cover the period from February 2015 to March 2016 for the intervention period as indicated in the table 6 below but the follow up after discharge will only be completed in March 2017.

Table 6: Study schedule

	Feb-15	Mar-15	Apr-15	May-15	Jun-15	Jul-15	Aug-15	Sep-15	~	Mar-16
Production										
Acceptability trial										
Production										
Efficacy trial										

2.13 Study Team

Valid: Paluku Bahwere, M.D PhD, Kate Sadler, PhD., Peter Akomo, Msc., Theresa Banda, Msc, Bsc.,. and Steve Collins, M.D, PhD.
Ajinomoto co: Hitoshi Murakami, PhD.
Free University of Brussels: Paluku Bahwere, M.D, PhD., Michèle Dramaix, PhD.
Ministry of Health Malawi: Sylvester Kathumba, Msc., Mwayi Mwale, M.D., Salomon Jere, M.D., Chimwemwe Banda M.D.
Ministry of Gender and Social welfare: McKnight Synos Hanniford Kalanda, M.A

The principal investigator has large experience in conducting randomized controlled trial including in Malawi where he was based for four years supporting as Valid representative supporting the Ministry of Health in piloting the community based management of acute malnutrition approach and running research to improve the approach and to expand the model to adults especially People living with HIV and AIDS. Several recent publications confirm his expertise in the area of community based management of acute malnutrition and of testing ready-to-use foods[11, 15, 16, 28, 34, 36, 92, 99-103].

2.14 Source of funding

This study is funded by Ajinomoto Co.,INC..

Reference List

1. Black RE, Allen LH, Bhutta ZA, Caulfield LE, de Onis M, Ezzati M, *et al.* Maternal and child undernutrition: global and regional exposures and health consequences. *Lancet* 2008; **371(9608)**:243-260.

2. Collins S, Dent N, Binns P, Bahwere P, Sadler K, Hallam A. Management of severe acute malnutrition in children. *Lancet* 2006; **368(9551)**:1992-2000.

3. Ubesie AC, Ibeziako NS, Ndiokwelu CI, Uzoka CM, Nwafor CA. Under-five Protein Energy Malnutrition Admitted at the University of In Nigeria Teaching Hospital, Enugu: a 10 year retrospective review. *Nutrition journal* 2012; **11(1)**:43.

4. Schofield C, Ashworth A. Why have mortality rates for severe malnutrition remained so high? *Bull World Health Organ* 1996; **74(2)**:223-229.

5. WHO, UNICEF, SCN. Joint statement on the community-based management of severe malnutrition in children. In: 2007.

6. Collins S. Changing the way we address severe malnutrition during famine. *Lancet* 2001; **358(9280)**:498-501.

7. Collins S, Sadler K. Outpatient care for severely malnourished children in emergency relief programmes: a retrospective cohort study. *Lancet* 2002; **360(9348)**:1824-1830.

8. Collins S. Community-based therapeutic care: A new paradigm for selective feeding in nutritional crises. In. London: Humanitarian Practice Network, Overseas Development Institute; 2004.

9. WHO. Report of an informal consultation on the community-based management of severe malnutrition in children. In: 2006.

10. Collins S, Sadler K, Dent N, Khara T, Guerrero S, Myatt M, *et al.* Key issues in the success of community-based management of severe malnutrition. *Food Nutr Bull* 2006; **27(3)**:S49-S82.

11. Bahwere P, Piwoz E, Joshua MC, Sadler K, Grobler-Tanner CH, Guerrero S, *et al.* Uptake of HIV testing and outcomes within a Community-based Therapeutic Care (CTC) programme to treat severe acute malnutrition in Malawi: a descriptive study. *BMC Infect Dis* 2008; **8**:106.

12. Ciliberto MA, Sandige H, Ndekha MJ, Ashorn P, Briend A, Ciliberto HM, *et al.* Comparison of home-based therapy with ready-to-use therapeutic food with standard therapy in the treatment of malnourished Malawian children: a controlled, clinical effectiveness trial. *Am J Clin Nutr* 2005; **81(4)**:864-870.

13. Ciliberto MA, Manary MJ, Ndekha MJ, Briend A, Ashorn P. Home-based therapy for oedematous malnutrition with ready-to-use therapeutic food. *Acta Paediatr* 2006; **95(8)**:1012-1015.

14. Rossi L, Hoerz T, Thouvenot V, Pastore G, Michael M. Evaluation of health, nutrition and food security programmes in a complex emergency: the case of Congo as an example of a chronic post-conflict situation. *Public Health Nutr* 2006; **9(5)**:551-556.

15. Bahwere P, Banda T, Sadler K, Nyirenda G, Owino V, Shaba B, *et al*. Effectiveness of milk whey protein-based ready-to-use therapeutic food in treatment of severe acute malnutrition in Malawian under-5 children: a randomised, double-blind, controlled non-inferiority clinical trial. *Matern Child Nutr* 2014.

16. Bahwere P, Mtimuni A, Sadler K, Banda T, Collins S. Long term mortality after community and facility based treatment of severe acute malnutrition: Analysis of data from Bangladesh, Kenya, Malawi and Niger. *Journal of Public Health and Epidemiology* 2012; **4(8)**:215-225.

17. Maleta K, Amadi B. Community-based management of acute malnutrition (CMAM) in sub-Saharan Africa: Case studies from Ghana, Malawi, and Zambia. *Food & Nutrition Bulletin* 2014; **35(Supplement 1)**:34S-38S.

18. Puett C, Sadler K, Alderman H, Coates J, Fiedler JL, Myatt M. Cost-effectiveness of the community-based management of severe acute malnutrition by community health workers in southern Bangladesh. *Health Policy Plan* 2012.

19. Puett C, Coates J, Alderman H, Sadler K. Quality of care for severe acute malnutrition delivered by community health workers in southern Bangladesh. *Matern Child Nutr* 2013; **9(1)**:130-142.

20. Dale NM, Myatt M, Prudhon C, Briend A. Using mid-upper arm circumference to end treatment of severe acute malnutrition leads to higher weight gains in the most malnourished children. *PloS one* 2013; **8(2)**:e55404.

21. Yebyo HG, Kendall C, Nigusse D, Lemma W. Outpatient therapeutic feeding program outcomes and determinants in treatment of severe acute malnutrition in tigray, northern ethiopia: a retrospective cohort study. *PLoS ONE* 2013; **8(6)**:e65840.

22. Dale NM, Myatt M, Prudhon C, Briend A. Using mid-upper arm circumference to end treatment of severe acute malnutrition leads to higher weight gains in the most malnourished children. *PLoS One* 2013; **8(2)**:e55404.

23. Briend A. Dietary management of severe protein-calorie malnutrition in children. *Ann Med Interne (Paris)* 2000; **151(8)**:629-634.

24. Briend A. Treatment of Severe Malnutrition with a Therapeutic Spread. *Field Exchange* 1997**(2)**:15.

25. Briend A, Lacsala R, Prudhon C, Mounier B, Grellety Y, Golden MHN. Ready-to-use therapeutic food for treatment of marasmus [letter]. *Lancet* 1999; **353(9166)**:1767-1768.

26. Manary MJ. Local production and provision of ready-to-use therapeutic food (RUTF) spread for the treatment of severe childhood malnutrition. *Food Nutr Bull* 2006; **27(3 Suppl)**:S83-S89.

27. World Health Organization. *Management of severe malnutrition : a manual for physicians and other senior health workers*: Geneva : World Health Organization, 1999; 1999.

28. Bahwere P. Severe acute malnutrition during emergencies: Burden, management, and gaps. *Food & Nutrition Bulletin* 2014; **35(Supplement 1)**:47S-51S.

29. Wagh VD, Deore BR. Ready to Use Therapeutic Food (RUTF): An Overview.

30. Diop EHI, Dossou NI, Ndour MM, Briend A, Wade S. Comparison of the efficacy of a solid ready to use food and a liquid milk-based diet for the rehabilitation of severely malnourished children: a randomized trial. *Am J Clin Nutr* 2003**(78)**:302-307.

31. Manary MJ, Ndkeha MJ, Ashorn P, Maleta K, Briend A. Home based therapy for severe malnutrition with ready-to-use food. *Arch Dis Child* 2004; **89(6)**:557-561.

32. Sandige H, Ndekha MJ, Briend A, Ashorn P, Manary MJ. Home-based treatment of malnourished Malawian children with locally produced or imported ready-to-use food. *J Pediatr Gastroenterol Nutr* 2004; **39(2)**:141-146.

33. Matumba L, Monjerezi M, Biswick T, Mwatseteza J, Makumba W, Kamangira D*, et al.* A survey of the incidence and level of aflatoxin contamination in a range of locally and imported processed foods on Malawian retail market. *Food Control* 2014; **39**:87-91.

34. Bahwere P, Sadler K, Collins S. Acceptability and effectiveness of chickpea sesame-based ready-to-use therapeutic food in malnourished HIV-positive adults. *Patient Preference and Adherence* 2009.

35. Irena A, Bahwere P, Owino VO, Diop EHI, Bachmann MO, Mbwili M*, et al.* Milk-free soy-maize-sorghum based ready-to-use therapeutic food is non-inferior to standard RUTF with 25% milk among severely acutely malnourished Zambian children; cluster randomized trial. In: 2012.

36. Owino VO, Bahwere P, Bisimwa G, Mwangi CM, Collins S. Breast-milk intake of 9-10-mo-old rural infants given a ready-to-use complementary food in South Kivu, Democratic Republic of Congo. *Am J Clin Nutr* 2011; **93(6)**:1300-1304.

37. Kumwenda C, Dewey KG, Hemsworth J, Ashorn P, Maleta K, Haskell MJ. Lipid-based nutrient supplements do not decrease breast milk intake of Malawian infants. *Am J Clin Nutr* 2014; **99(3)**:617-623.

38. Galpin L, Thakwalakwa C, Phuka J, Ashorn P, Maleta K, Wong WW*, et al.* Breast milk intake is not reduced more by the introduction of energy dense complementary food than by typical infant porridge

9. *J Nutr* 2007; **137(7)**:1828-1833.

39. Roos N, Sorensen JC, Sorensen H, Rasmussen SK, Briend A, Yang Z*, et al.* Screening for anti–nutritional compounds in complementary foods and food aid products for infants and young children. *Maternal & child nutrition* 2013; **9(S1)**:47-71.

40. Troesch B, Egli I, Zeder C, Hurrell RF, Zimmermann MB. Fortification iron as ferrous sulfate plus ascorbic acid is more rapidly absorbed than as sodium iron EDTA but neither increases serum nontransferrin-bound iron in women. *The Journal of Nutrition* 2011; **141(5)**:822-827.

41. Cercamondi CI, Egli IM, Mitchikpe E, Tossou F, Hessou J, Zeder C*, et al.* Iron bioavailability from a lipid-based complementary food fortificant mixed with millet porridge can be optimized by adding phytase and ascorbic acid but not by using a mixture of ferrous sulfate and sodium iron EDTA. *The Journal of Nutrition* 2013; **143(8)**:1233-1239.

42. Troesch B, van Stuijvenberg ME, Smuts CM, Kruger HS, Biebinger R, Hurrell RF*, et al.* A micronutrient powder with low doses of highly absorbable iron and zinc reduces iron and zinc deficiency and improves weight-for-age Z-scores in South African children. *J Nutr* 2011; **141(2)**:237-242.

43. Zimmermann MB, Chassard C, Rohner F, N'Goran EzK, Nindjin C, Dostal A, *et al.* The effects of iron fortification on the gut microbiota in African children: a randomized controlled trial in Cote d'Ivoire. *The American journal of clinical nutrition* 2010; **92(6)**:1406-1415.

44. Soofi S, Cousens S, Iqbal SP, Akhund T, Khan J, Ahmed I, *et al.* Effect of provision of daily zinc and iron with several micronutrients on growth and morbidity among young children in Pakistan: a cluster-randomised trial. *The Lancet* 2013; **382(9886)**:29-40.

45. Diop EHI, Dossou NI, Ndour MM, Briend A, Wade S. Hemoglobin variations during nutrition rehabilitation of severely malnourished children with a solid or liquid diet with different level of iron. In: 2003.

46. Ozkale M, Sipahi T. Hematologic and Bone Marrow Changes in Children with Protein-Energy Malnutrition. *Pediatric Hematology-Oncology* 2014; **31(4)**:349-358.

47. Glinz D, Kamiyango M, Phiri KS, Munthali F, Zeder C, Zimmermann MB, *et al.* The effect of timing of iron supplementation on iron absorption and haemoglobin in post-malaria anaemia: a longitudinal stable isotope study in Malawian toddlers. *Malaria journal* 2014; **13(1)**:397.

48. Phiri KS, Calis JC, Faragher B, Nkhoma E, Ng'oma K, Mangochi B, *et al.* Long term outcome of severe anaemia in Malawian children. *PLoS ONE* 2008; **3(8)**:e2903.

49. Branca F, Robins SP, Ferro-Luzzi A, Golden MHN. Bone turnover in malnourished children. *Lancet* 1992; **340**:1493-1496.

50. Fjeld CR, Schoeller DA, Brown KH. Body composition of children recovering from severe protein-energy malnutrition at two rates of catch-up growth. *Am J Clin Nutr* 1989; **50(6)**:1266-1275.

51. Radhakrishna KV, Kulkarni B, Balakrishna N, Rajkumar H, Omkar C, Shatrugna V. Composition of weight gain during nutrition rehabilitation of severely under nourished children in a hospital based study from India. *Asia Pac J Clin Nutr* 2010; **19(1)**:8-13.

52. Walker SP, Golden MH. Growth in length of children recovering from severe malnutrition. *Eur J Clin Nutr* 1988; **42(5)**:395-404.

53. Oakley E, Reinking J, Sandige H, Trehan I, Kennedy G, Maleta K, *et al.* A ready-to-use therapeutic food containing 10% milk is less effective than one with 25% milk in the treatment of severely malnourished children. *J Nutr* 2010; **140(12)**:2248-2252.

54. Brambilla P, Rolland-Cachera MF, Testolin C, Briend A, Salvatoni A, Testolin G, *et al.* Lean mass of children in various nutritional states. Comparison between dual-energy X-ray absorptiometry and anthropometry. *Ann N Y Acad Sci* 2000; **904**:433-436.

55. Brown KH, Black RE, Becker S. Seasonal changes in nutritional status and the prevalence of malnutrition in a longitudinal study of young children in rural Bangladesh. *Am J Clin Nutr* 1982; **36(2)**:303-313.

56. Maleta K, Virtanen SM, Espo M, Kulmala T, Ashorn P. Seasonality of growth and the relationship between weight and height gain in children under three years of age in rural Malawi. *Acta Paediatr* 2003; **92(4)**:491-497.

57. Isanaka S, Roederer T, Djibo A, Luquero FJ, Nombela N, Guerin PJ, *et al.* Reducing wasting in young children with preventive supplementation: a cohort study in Niger. *Pediatrics* 2010; **126(2)**:e442-e450.

58. Bartz S, Mody A, Hornik C, Bain J, Muehlbauer M, Kiyimba T, *et al.* Severe acute malnutrition in childhood: hormonal and metabolic status at presentation, response to treatment, and predictors of mortality. *The Journal of Clinical Endocrinology & Metabolism* 2014.

59. Bartz S, Mody A, Hornik C, Bain J, Muehlbauer M, Kiyimba T, *et al.* Severe acute malnutrition in childhood: hormonal and metabolic status at presentation, response to treatment, and predictors of mortality. *The Journal of Clinical Endocrinology & Metabolism* 2014.

60. Keusch GT, Rosenberg IH, Denno DM, Duggan C, Guerrant RL, Lavery JV, *et al.* Implications of acquired environmental enteric dysfunction for growth and stunting in infants and children living in low- and middle-income countries. *Food Nutr Bull* 2013; **34(3)**:357-364.

61. Humphrey JH. Child undernutrition, tropical enteropathy, toilets, and handwashing. *Lancet* 2009; **374(9694)**:1032-1035.

62. Haghighi P, Wolf PL. Tropical sprue and subclinical enteropathy: a vision for the nineties. *Crit Rev Clin Lab Sci* 1997; **34(4)**:313-341.

63. Lunn PG, Northrop-Clewes CA, Downes RM. Intestinal permeability, mucosal injury, and growth faltering in Gambian infants. *Lancet* 1991; **338(8772)**:907-910.

64. Ngure FM, Reid BM, Humphrey JH, Mbuya MN, Pelto G, Stoltzfus RJ. Water, sanitation, and hygiene (WASH), environmental enteropathy, nutrition, and early child development: making the links. *Ann N Y Acad Sci* 2014; **1308**:118-128.

65. Prendergast AJ, Rukobo S, Chasekwa B, Mutasa K, Ntozini R, Mbuya MN, *et al.* Stunting is characterized by chronic inflammation in Zimbabwean infants. *PLoS One* 2014; **9(2)**:e86928.

66. Smith MI, Yatsunenko T, Manary MJ, Trehan I, Mkakosya R, Cheng J, *et al.* Gut microbiomes of Malawian twin pairs discordant for kwashiorkor. *Science* 2013; **339(6119)**:548-554.

67. Subramanian S, Huq S, Yatsunenko T, Haque R, Mahfuz M, Alam MA, *et al.* Persistent gut microbiota immaturity in malnourished Bangladeshi children. *Nature* 2014; **510(7505)**:417-421.

68. Bin-Nun A, Booms C, Sabag N, Mevorach R, Algur N, Hammerman C. Rapid Fecal Calprotectin (FC) Analysis: Point of Care Testing for Diagnosing Early Necrotizing Enterocolitis. *American journal of perinatology* 2014**(EFirst)**.

69. Hippe B, Remely M, Bartosiewicz N, Riedel M, Nichterl C, Schatz L, *et al.* Abundance and Diversity of GI Microbiota Rather than IgG4 Levels Correlate with Abdominal Inconvenience and Gut Permeability in Consumers Claiming Food Intolerances. *Endocrine, Metabolic & Immune Disorders-Drug Targets (Formerly Current Drug Targets-Immune, Endocrine & Metabolic Disorders)* 2014; **14(1)**:67-75.

70. Bachmann MO. Cost effectiveness of community-based therapeutic care for children with severe acute malnutrition in Zambia: decision tree model. *Cost Eff Resour Alloc* 2009; **7**:2.

71. Bachmann MO. Cost-effectiveness of community-based treatment of severe acute malnutrition in children. *Expert Rev Pharmacoecon Outcomes Res* 2010; **10(5)**:605-612.

72. Jha P, Bangoura O, Ranson K. The cost-effectiveness of forty health interventions in Guinea. *Health Policy Plan* 1998; **13(3)**:249-262.

73. Tekeste A, Wondafrash M, Azene G, Deribe K. Cost effectiveness of community-based and in-patient therapeutic feeding programs to treat severe acute malnutrition in Ethiopia. *Cost Eff Resour Alloc* 2012; **10**:4.

74. Wilford R, Golden K, Walker DG. Cost-effectiveness of community-based management of acute malnutrition in Malawi. *Health Policy Plan* 2012; **27(2)**:127-137.

75. Roosmalen-Wiebenga MW, Kusin JA, de With C. Nutrition rehabilitation in hospital--a waste of time and money? Evaluation of nutrition rehabilitation in a rural district hospital in southwest Tanzania. I. Short-term results. *J Trop Pediatr* 1986; **32(5)**:240-243.

76. Roosmalen-Wiebenga MW, Kusin JA, de With C. Nutrition rehabilitation in hospital--a waste of time and money? Evaluation of nutrition rehabilitation in a rural district hospital in South-west Tanzania. II. Long-term results. *J Trop Pediatr* 1987; **33(1)**:24-28.

77. Cook R. The financial cost of malnutrition in the "Commonwealth Caribbean". *J Trop Pediatr* 1968; **14(2)**:60-65.

78. Cook R. Is hospital the place for the treatment of malnourished children? *J Trop Pediatr Environ Child Health* 1971; **17(1)**:15-25.

79. Ashworth A. Efficacy and effectiveness of community-based treatment of severe malnutrition. *Food Nutr Bull* 2006; **27(3 Suppl)**:S24-S48.

80. Chapko MK, Prual A, Gamatie Y, Maazou AA. Randomized clinical trial comparing hospital to ambulatory rehabilitation of malnourished children in Niger. *J Trop Pediatr* 1994; **40(4)**:225-230.

81. Pecoul B, Soutif C, Hounkpevi M, Ducos M. Efficacy of a therapeutic feeding centre evaluated during hospitalization and a follow-up period, Tahoua, Niger, 1987-1988. *Ann Trop Paediatr* 1992; **12(1)**:47-54.

82. Reneman L, Derwig J. Long-term prospects of malnourished children after rehabilitation at the Nutrition Rehabilitation Centre of St Mary's Hospital, Mumias, Kenya. *J Trop Pediatr* 1997; **43(5)**:293-296.

83. Khanum S, Ashworth A, Huttly SR. Growth, morbidity, and mortality of children in Dhaka after treatment for severe malnutrition: a prospective study. *Am J Clin Nutr* 1998; **67(5)**:940-945.

84. Bahwere P, Mtimuni A, Sadler K, Banda T, Collins S. Long term mortality after community and facility based treatment of severe acute malnutrition: Analysis of data from Bangladesh, Kenya. Malawi and Niger. *Journal of Public Health and Epidemiology* 2012; **4(8)**:215-225.

85. Beesabathuni KN, Natchu UC. Production and distribution of a therapeutic nutritional product for severe acute malnutrition in India: opportunities and challenges. *Indian Pediatr* 2010; **47(8)**:702-706.

86. Dube B, Rongsen T, Mazumder S, Taneja S, Rafiqui F, Bhandari N, *et al.* Comparison of Ready-to-Use Therapeutic Food with cereal legume-based khichri among malnourished children. *Indian Pediatr* 2009; **46(5)**:383-388.

87. Khlangwiset P, Shephard GS, Wu F. Aflatoxins and growth impairment: a review. *Crit Rev Toxicol* 2011; **41(9)**:740-755.

88. Yang Z. Are peanut allergies a concern for using peanut-based formulated foods in developing countries? *Food Nutr Bull* 2010; **31(2 Suppl)**:S147-S153.

89. Briend A, Prinzo ZW. Dietary management of moderate malnutrition: time for a change. *Food Nutr Bull* 2009; **30(3 Suppl)**:S265-S266.

90. Owino VO, Irena AH, Dibari F, Collins S. Development and acceptability of a novel milk-free soybean-maize-sorghum ready-to-use therapeutic food (SMS-RUTF) based on industrial extrusion cooking process. *Matern Child Nutr* 2012.

91. Irena A, Bahwere P, Owino VO, Diop el HI, Bachmann MO, Mbwili-Muleya C, *et al.* Comparison of the effectiveness of a milk-free soy-maize-sorghum based ready-to-use therapeutic food to standard ready-to-use therapeutic food with 25% milk in nutrition management of severely acutely malnourished Zambian children: An equivalence non-blinded cluster randomized controlled trial. In: 2012.

92. Irena AH, Bahwere P, Owino VO, Diop EI, Bachmann MO, Mbwili-Muleya C, *et al.* Comparison of the effectiveness of a milk-free soy-maize-sorghum-based ready-to-use therapeutic food to standard ready-to-use therapeutic food with 25% milk in nutrition management of severely acutely malnourished Zambian children: an equivalence non-blinded cluster randomised controlled trial. *Matern Child Nutr* 2013.

93. National Statistical Office (NSO), ICF Macro.2011. Malawi Demographic and Health Survey 2010. In. Zomba, Malawi and Calverton, Maryland, USA: NSO and ICF Macro.; 2011.

94. Blackwelder WC. "Proving the null hypothesis" in clinical trials. *Control Clin Trials* 1982; **3(4)**:345-353.

95. Julious SA. Sample sizes for clinical trials with normal data. *Stat Med* 2004; **23(12)**:1921-1986.

96. Coward WA, Cole TJ, Sawyer MB, Prentice AM. Breast-milk intake measurement in mixed-fed infants by administration of deuterium oxide to their mothers. *Hum Nutr Clin Nutr* 1982; **36(2)**:141-148.

97. World Health Organization. WHO guidelines on drawing blood: best practices in phlebotomy. 2010.

98. Piaggio G, Elbourne DR, Altman DG, Pocock SJ, Evans SJ. Reporting of noninferiority and equivalence randomized trials: an extension of the CONSORT statement. *JAMA* 2006; **295(10)**:1152-1160.

99. Bahwere P, Deconinck H, Banda T, Mtimuni A, Collins S. Impact of household food insecurity on the nutritional status and the response to therapeutic feeding of people living with human immunodeficiency virus. *Patient Prefer Adherence* 2011; **5**:619-627.

100. Bisimwa G, Owino VO, Bahwere P, Dramaix M, Donnen P, Dibari F, *et al.* Randomized controlled trial of the effectiveness of a soybean-maize-sorghum-based ready-to-use complementary food paste on infant growth in South Kivu, Democratic Republic of Congo. *Am J Clin Nutr* 2012; **95(5)**:1157-1164.

101. Dibari F, Bahwere P, Le G, I, Guerrero S, Mwaniki D, Seal A. A qualitative investigation of adherence to nutritional therapy in malnourished adult AIDS patients in Kenya. *Public Health Nutr* 2011:1-8.

102. Dibari F, Bahwere P, Le G, I, Guerrero S, Mwaniki D, Seal A. A qualitative investigation of adherence to nutritional therapy in malnourished adult AIDS patients in Kenya. *Public Health Nutr* 2012; **15(2)**:316-323.

103. Dibari F, Bahwere P, Huerga H, Irena AH, Owino V, Collins S, *et al*. Development of a cross-over randomized trial method to determine the acceptability and safety of novel ready-to-use therapeutic foods. *Nutrition* 2013; **29(1)**:107-112.
